# AB_5_ Derivatives of Cyclotriphosphazene for the Synthesis of Dendrons and Their Applications

**DOI:** 10.3390/molecules26134017

**Published:** 2021-06-30

**Authors:** Artem Zibarov, Abdelouahd Oukhrib, Julien Aujard Catot, Cédric-Olivier Turrin, Anne-Marie Caminade

**Affiliations:** 1 Laboratoire de Chimie de Coordination du CNRS, 205 Route de Narbonne, 31077 Toulouse, CEDEX 4, France; artem.zibarov@lcc-toulouse.fr (A.Z.); abdelouahd.oukhrib@lcc-toulouse.fr (A.O.); julien.aujard@lcc-toulouse.fr (J.A.C.); cedric-olivier.turrin@lcc-toulouse.fr (C.-O.T.); 2 LCC-CNRS, Université de Toulouse, CNRS, 31077 Toulouse, CEDEX 4, France

**Keywords:** cyclotriphosphazene, dendron, fluorescence, phosphorus, dendrimer, nanomaterials, sensors, catalysis, nanomedicine

## Abstract

AB_5_ compounds issued from the reactivity of hexachlorocyclotriphosphazene are relatively easy to obtain using two ways: either first the reaction of one chloride with one reagent, followed by the reaction of the five remaining Cl with another reagent, or first the reaction of five chlorides with one reagent, followed by the reaction of the single remaining Cl with another reagent. This particular property led to the use of such compounds as core for the synthesis of dendrons (dendritic wedges), using the five functions for growing the dendritic branches. The single function can be used for the synthesis of diverse types of dendrimers (onion peel, dumbbell-shape, Janus), for covalent or non-covalent grafting to solid surfaces, providing nanomaterials, for grafting a fluorophore, especially for studying biological mechanisms, or for self-associations to get micelles. All these properties are reviewed in this paper.

## 1. Introduction

**Scheme 1:**
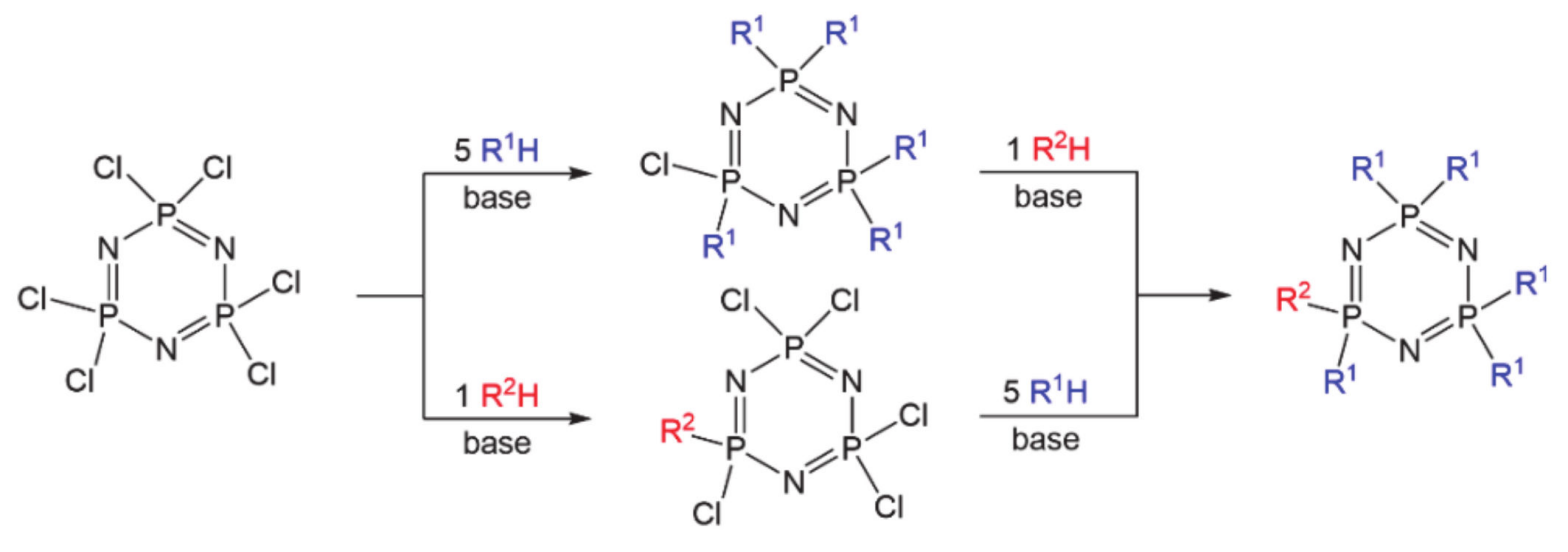
Two ways for the synthesis of AB_5_ derivatives of cyclotriphosphazene.

Hexachlorocyclotriphosphazene (N=PCl_2_)_3_, also written N_3_P_3_Cl_6_, was synthesized a long time ago, in the first part of the 19th century [1], but correctly analyzed more than 30 years later [2]. Since that time, a huge number of reactions have been carried out, essentially of two types, either the thermal ring opening leading to polyphosphazenes [3], or nucleophilic substitutions of all the chlorides, most generally with alcohols/phenols or amines. An interesting feature of N_3_P_3_Cl_6_ is the possibility to differentiate the reactivity of one chlorine from the five others. Two different ways have been used to perform such differentiation: either one Cl reacts with one reagent, then the remaining five Cl react with another reagent, or 5 Cl react with one reagent, then the single remaining Cl reacts with another reagent. Both ways to obtain AB_5_ derivatives of N_3_P_3_ are shown in Scheme 1. The desired compounds are generally obtained in good yield after column chromatography.

Finding the best way to obtain the desired AB_5_ or A_5_B derivative, as shown in Scheme 1, is still an open question. Indeed, there are very few publications comparing the efficiency of these two ways, and results are often contradictory. The only clear answer is when one of the reagents bears a function that is not compatible (not orthogonal) with the reaction used for grafting the second reagent. The most sensitive reagent should be used in the second step, using either one or five equivalents, depending on the final desired compound. However, the number of references concerning several types of AB_5_ (or A_5_B) derivatives of cyclotriphosphazene (searched on Scifinder^®^) demonstrates that the type of substitution reaction influences the preferred way. In addition, oxygen and nitrogen derivatives are widely used, whereas carbon derivatives are relatively rare. For reactions with amines or with carbon derivatives, it is largely preferred to replace first one chloride instead of five (83.5% for one N and 79.8% for one C) on N_3_P_3_Cl_6_. The situation is more contrasted when using oxygen derivatives (frequently phenols) as the percentage of publications displaying a single reaction is 61.4% (Table 1).

The early experiences concerning the differentiation of the reactivity of the hexachlorocyclotriphosphazene have explored the possibility to selectively react one to six chlorides, in most cases using primary or, eventually, secondary amines. Such types of work were carried out, for instance, with dimethylamine [4], *ter*-butylamine [5], or isopropylamine [6]. The monosubstituted derivatives were isolated and characterized in all cases, as well as several di-, tri-, tetra-substituted derivatives, and the hexa-substituted derivatives, but not the penta-substituted ones. Other early examples of reactivity concerned enolate anions of acetaldehyde, acetone, and acetophenone, using lithium derivatives [7], methylsilane and methylsiloxane, using Grignard reagents [8], and phenols, using the sodium salt [9]. In the latter case, the penta-substituted derivative was easily obtained, and frequently used.

**Scheme 2:**
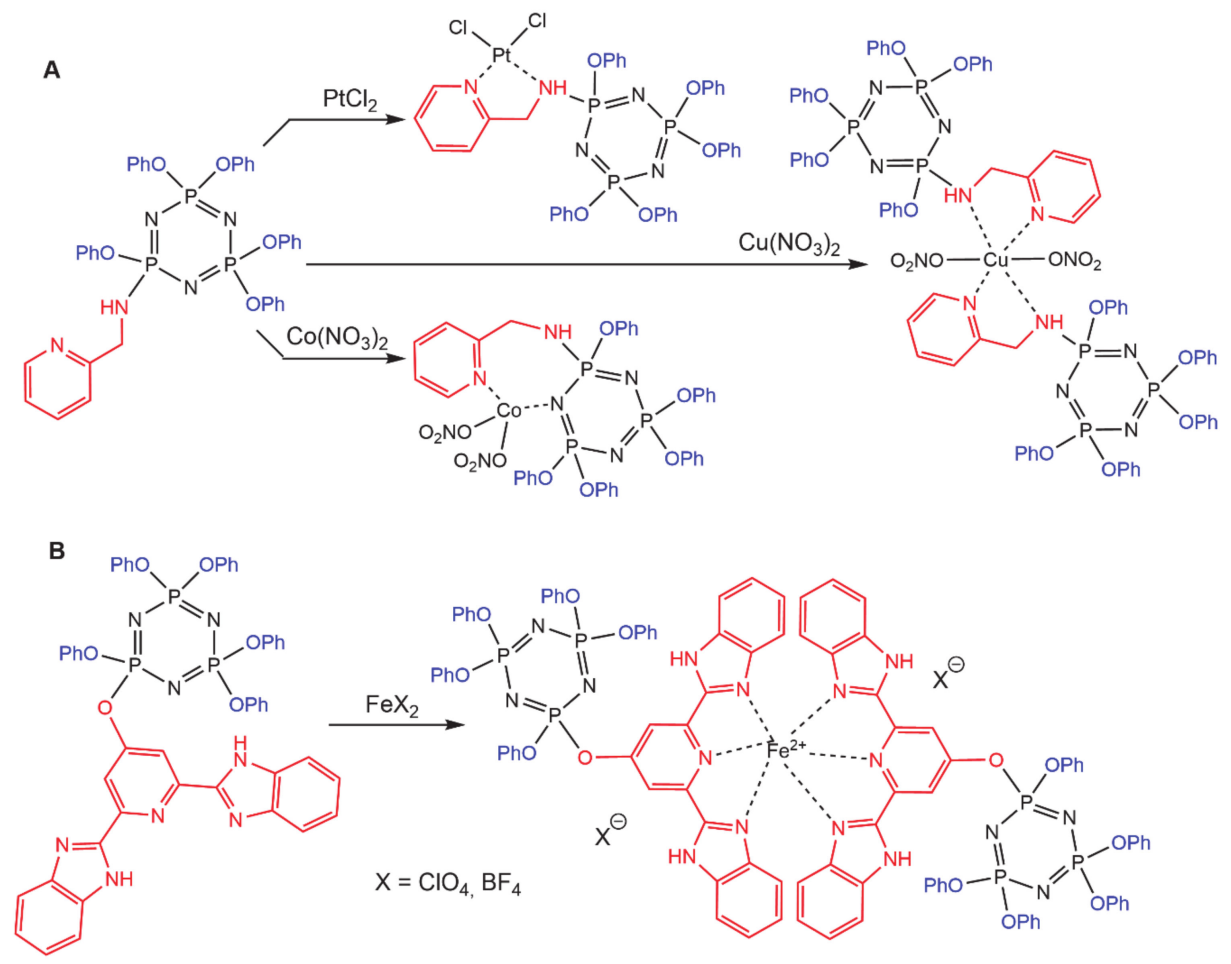
Examples of complexations with AB_5_ derivatives of cyclotriphosphazene, with either one or two ligands. (**A**) 2-pyridylmethylamine ligand. (**B**) 2,6-bis(benzimidazole-2-yl)pyridine ligand.

More recent results display a few practical uses of AB_5_ derivatives of cyclotriphosphazene, concerning in particular complexation properties, and polymerizations. The penta-phenoxy derivative was functionalized with a single 2-pyridylmethylamine ligand, then this compound was used for the complexation of Cu(NO_3_)_2_, PtCl_2_, and Co(NO_3_)_2_, affording a different type of complex in each case. As shown in Scheme 2A, platinum and copper complexes are monomeric, albeit different, whereas the copper complex contains two ligands [10]. Another example of complexation concerned also the penta-phenoxy derivative, functionalized with a single 2,6-bis(benzimidazole-2-yl)pyridine ligand. The reaction with FeX_2_ (X = ClO_4_^−^ or BF_4_^−^) afforded complexes bearing two ligands around iron, as shown in Scheme 2B. These complexes are low spin below 300 K, but display spin crossover (SCO) behavior above this temperature [11].

**Scheme 3:**
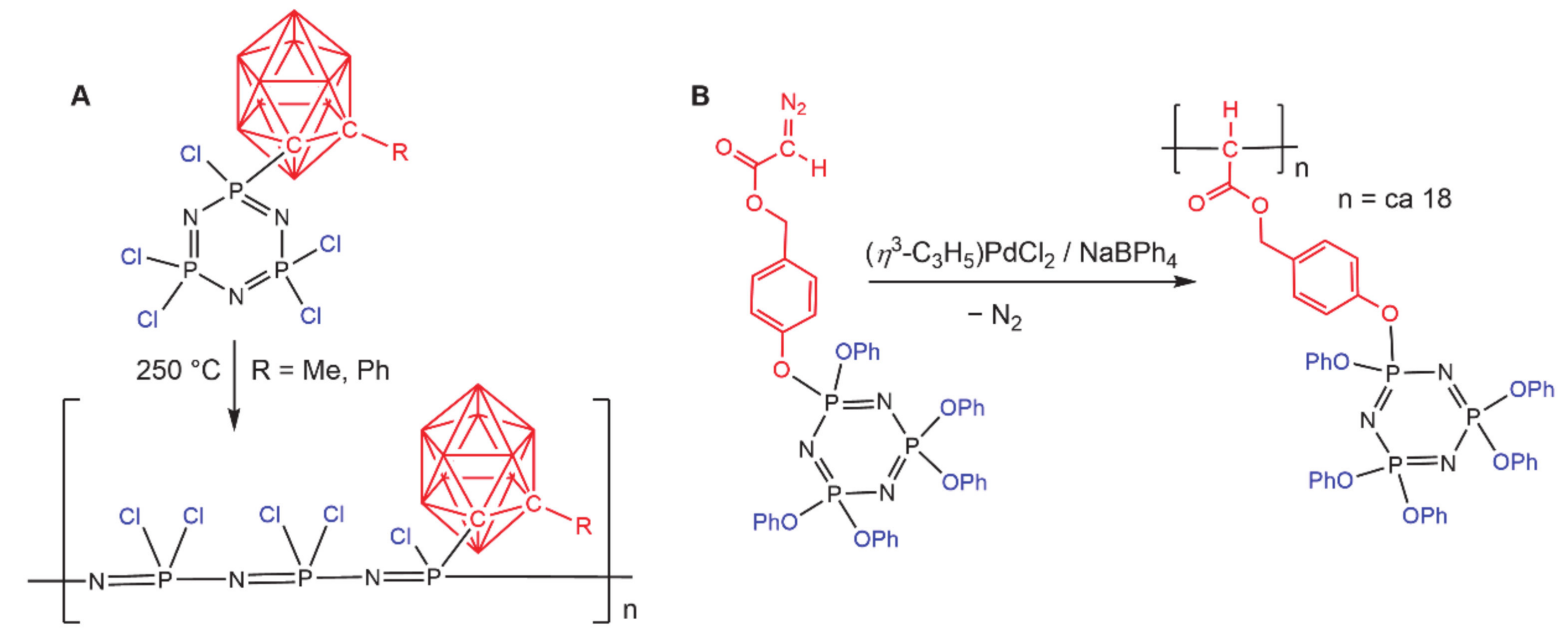
Examples of polymerization reaction of AB_5_ derivatives of cyclotriphosphazene. (**A**) Polymerization of N_3_P_3_. (**B**) Polymerization of a substituent of N_3_P_3_.

As indicated at the beginning of this introduction, cyclotriphosphazenes are widely used as precursors of polyphosphazenes. A few AB_5_ derivatives of cyclotriphosphazene have been used for such purpose. A carborane-substituted cyclotriphosphazene, obtained by reacting a lithiocarborane with N_3_P_3_Cl_6_, was polymerized when heated at 250 °C for 120 h (Scheme 3A). The Cl of the polyphosphazene were then replaced by reaction with sodium trifluoroethoxide, to avoid the hydrolytic instability of polydichlorophosphazenes [12,13]. A recent example of polymerization involved a substituent of the cyclotriphosphazene, thus it became a pendant group of the polymer. The pentaphenoxy derivative was functionalized with a single diazoacetate, which was polymerized, as shown in Scheme 3B, in the presence of a palladium catalyst [14].

**Figure 1:**
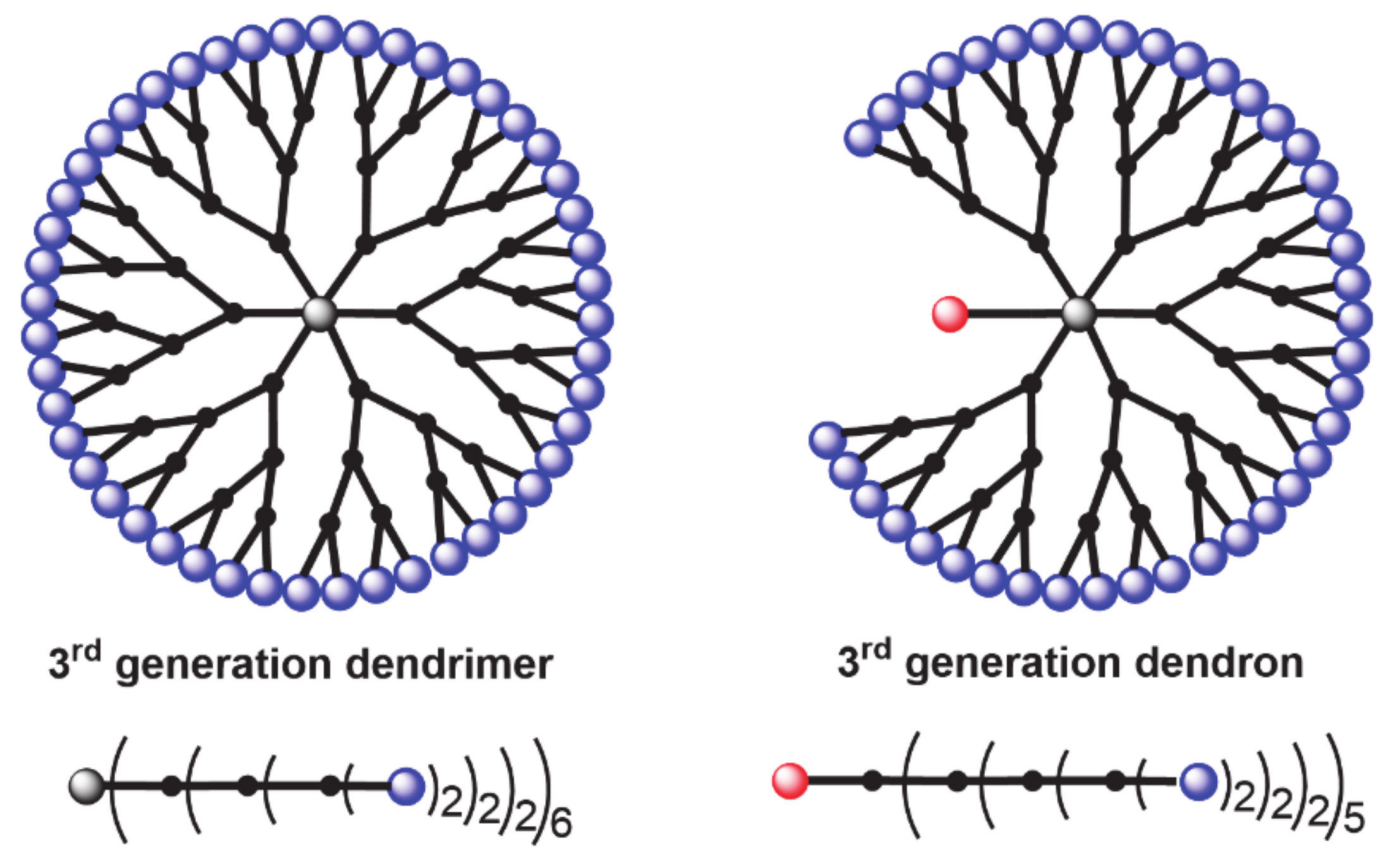
Schematized drawing of third generation dendrimer and dendron, built from an hexafunctional core such as N_3_P_3_. Full structure, and linear structure with parentheses.

Being interested since a long time in the field of dendritic structures [15] and their applications [16], we have widely used hexa-substituted cyclotriphosphazene as core of dendrimers [17], but also in a few cases AB_5_ derivatives of cyclotriphosphazene as building block for the construction of dendrimers. Dendrimers are hyperbranched macromolecules, synthesized step-by-step from a multifunctional core, by an iterative process, which generates a new “generation” each time the number of terminal functions is multiplied. Another family of dendritic molecules is called “dendrons” when one function at the level of the core is different from the others, which are used for the growing of the branches. Dendrimers and dendrons, both of third generation, are schematized in Figure 1. They are represented as two-dimensional structures, but they have three-dimensional shapes. In the lower part of Figure 1 is represented another way to draw dendrimers and dendrons, i.e., a linear form, with parentheses after each layer of branching points, with a number indicating the multiplicity at the considered layer. Multiplication of all these numbers gives the number of terminal functions (2 × 2 × 2 × 6) = 48 for the dendrimer, 2 × 2 × 2 × 5 = 40 for the dendron, as shown in Figure 1.

This review will be divided in four parts. The first part will show AB_5_ derivatives of N_3_P_3_, including small dendrons, used for the synthesis of diverse types of dendrimers, including layered, dumbbell-shape and Janus dendrimers. The second part will concern the covalent or non-covalent grafting to diverse surfaces of dendrons based on a N_3_P_3_ core. The third part will concern the use in biology of fluorescent dendrons based on a N_3_P_3_ core. The fourth part will display other biological uses of dendrons based on a N_3_P_3_ core.

## 2. AB_5_ Derivatives of N_3_P_3_ as Core and Their Use for the Synthesis of Various Types of Dendrimers

A few examples of dendrimers having AB_5_ derivatives of N_3_P_3_ as branching points are known. In a first case, the synthesis consisted in reacting N_3_P_3_Cl_6_ used as core with a diamine. The second step was the grafting of N_3_P_3_Cl_6_ on each terminal function. Then, the repetition of the first step was the reaction of the diamine with the five theoretically-remaining Cl on the cyclotriphosphazene [18]. However, the use at each step of reagents having two (diamine) or six (N_3_P_3_Cl_6_) identical functions precluded the obtaining of pure compounds.

### 2.1. Layered “Onion-Peel” Dendrimers

**Scheme 4:**
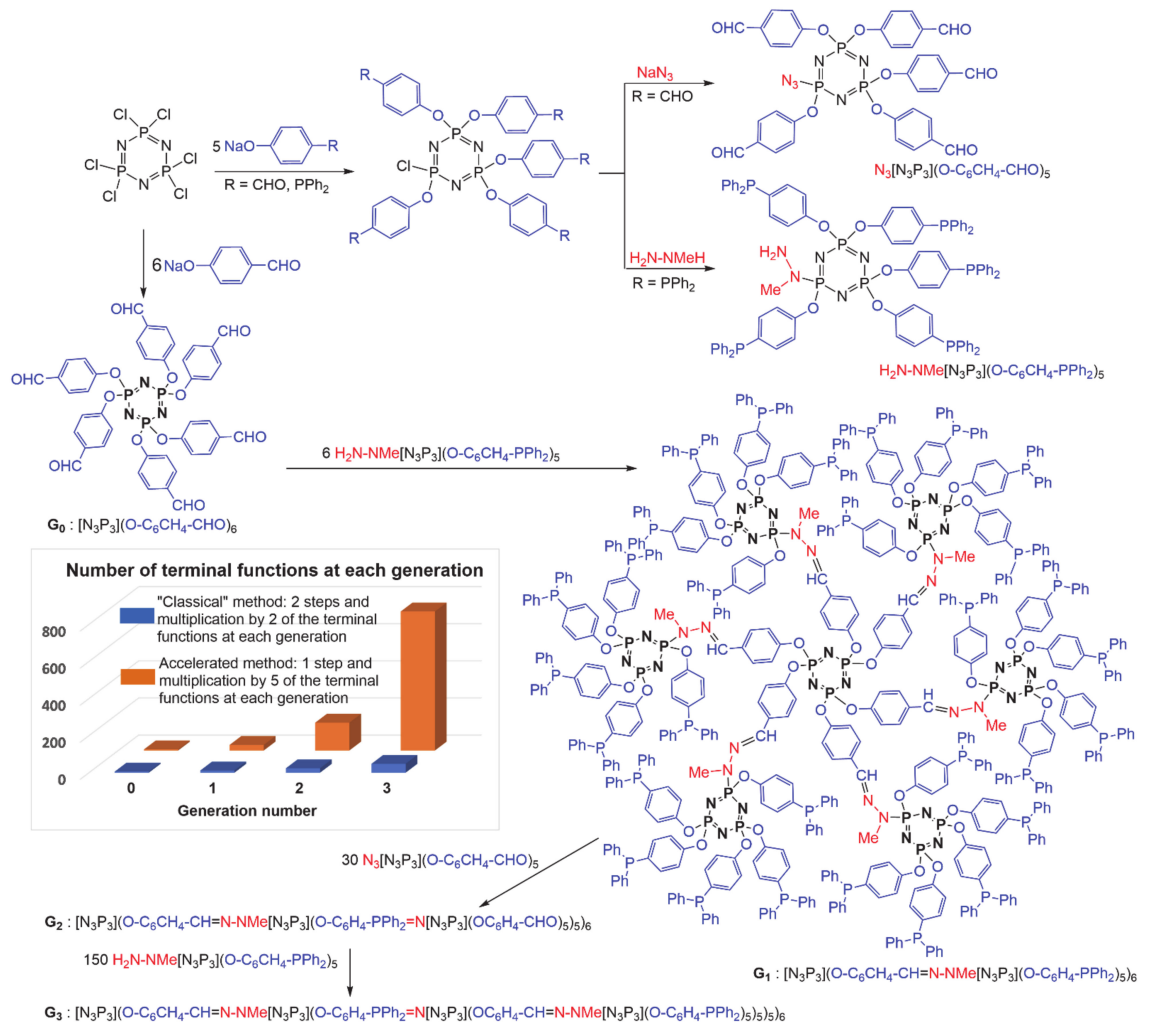
Two examples of AB_5_ derivatives of cyclotriphosphazene, used for the synthesis of layered phosphorus dendrimers. Full structure of generations 0 and 1, and linear structure of the four generations of the dendrimers, from **G_0_** to **G_3_**, having one, two, three or four layers of cyclotriphosphazenes. The graph represents the number of terminal functions at each generation, depending on the method of synthesis: widely used methods needing two steps for each generation and a multiplication by 2 at each generation (dark blue), or the accelerated method shown in this Scheme, one step for each generation, and a multiplication by 5 at each generation (orange).

For an accelerated synthesis of layered dendrimers having a precisely defined structure, two different types of AB_5_ derivatives of cyclotriphosphazene were synthesized, bearing five aldehydes and one azide or five phosphines and one hydrazine. Both compounds were synthesized by reacting first five phenol derivatives, then either one azide or one methylhydrazine, as shown in Scheme 4. Starting from a hexaaldehyde core (generation 0), the hydrazine derivative was condensed to afford a first-generation dendrimer functionalized with 30 phosphines. In a second step, the azide derivative was reacted with the phosphine dendrimer in a Staudinger reaction producing P=N linkages, to afford a second-generation dendrimer, bearing 150 aldehyde terminal functions. The third and last step of the synthesis process concerned again the condensation of the hydrazine derivative with the aldehyde terminal functions, to afford the third-generation dendrimer functionalized with 750 phosphines [19]. This compound is the largest dendrimer ever synthesized in only three steps. The full structure of **G_0_** and **G_1_**, as well as the linear structures of the four generations (**G_0_** to **G_3_**) of this family of dendrimer is also shown in Scheme 4.

**Scheme 5:**
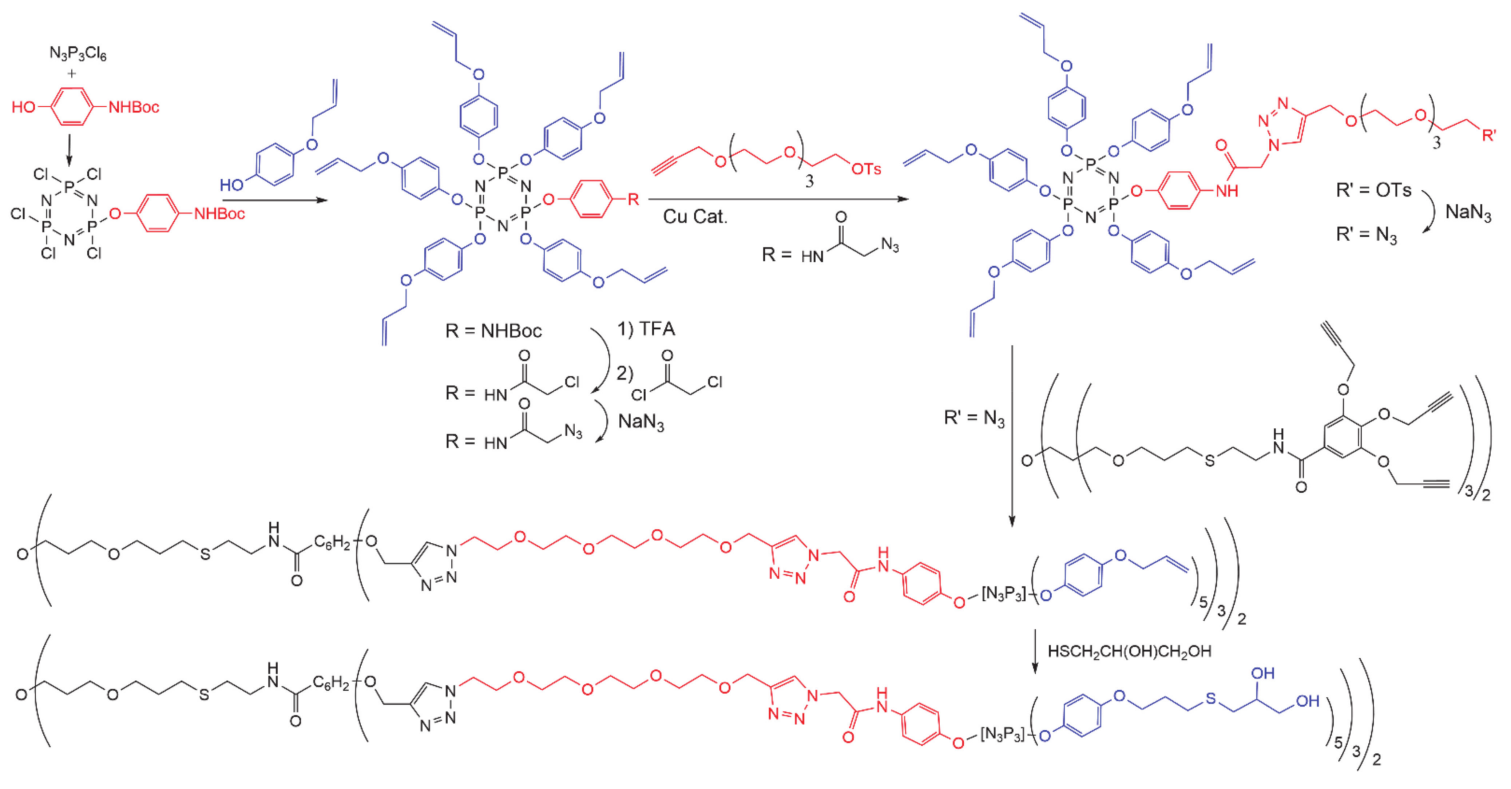
“Onion peel” (layered) dendrimers having one layer of cyclotriphosphazenes (N_3_P_3_), and synthesized via click reactions between alkynes and azides.

More recently, the cyclotriphosphazene was used for the synthesis of another type of “onion peel” (layered) dendrimers. The cyclotriphosphazene was first functionalized with a single *N*-Boc-protected 4-aminophenol, then with five p-allyloxyphenol. *N*-Boc-deprotection and subsequent *N*-chloroacetylation with chloroacetyl chloride and Hunig’s base provided a chloride function as substituent of the core, which was further substituted by an azide group using NaN_3_. The azide was then reacted with monopropargylated tosyltetraethylene glycol under classical “click” reaction conditions, also named Huisgen (3 + 2) cycloaddition, between an azide and an alkyne in the presence of a copper catalyst, affording a triazole [20]. Such a reaction has been frequently used for the synthesis of dendrimers [21]. Then, the tosyl group was substituted with an azide group using NaN_3_ (Scheme 5). The azide function of this AB_5_ derivative was then grafted to an octadecavalent hypercore equipped with 18 propargyl groups, again via a click reaction. The resulting dendrimer had 90 allyl terminal groups, which were finally reacted with 1-thioglycerol, to afford a dendritic structure functionalized with 180 alcohol groups [22].

**Scheme 6:**
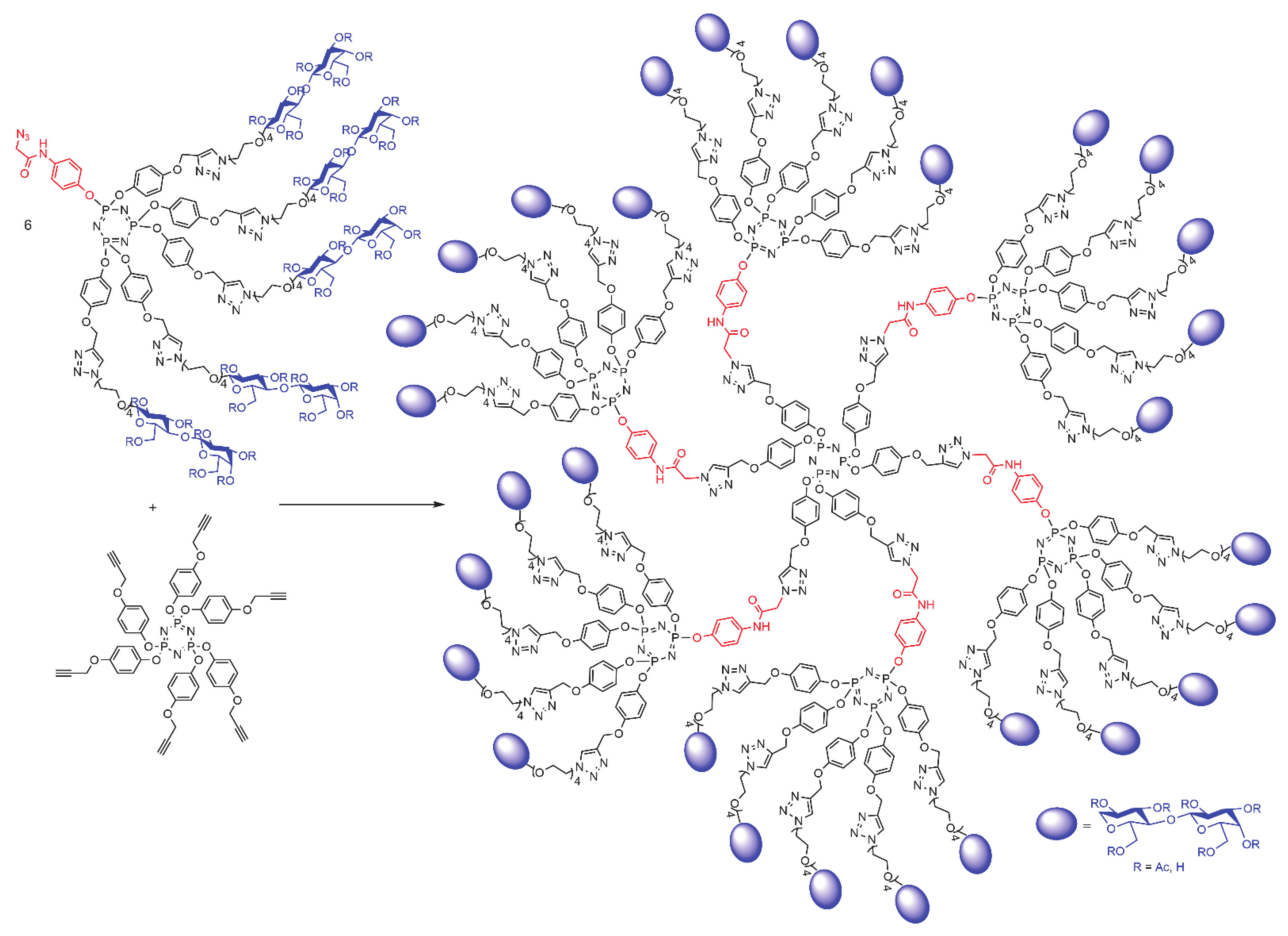
Synthesis of a layered onion peel dendrimer by click chemistry, starting from a dendron bearing lactoside terminal functions. The deprotection of the carbohydrate is carried out in the last step.

An extension of this work was carried out to get a series of dendritic molecules bearing lactoside terminal functions, starting in particular from the dendron shown in Scheme 6. Diverse examples of layered dendrimers were synthesized (one typical example of first-generation layered dendrimer is shown in Scheme 6). The synthesis was carried out in all cases with the acetylated carbohydrates, which were deprotected only in the final step. The largest compound of this family of onion peel dendrimers contained up to 90 lactose moieties. The biological activity of these compounds was studied with a bacterial virulence factor, and human adhesion/growth-regulatory lectin, and showed multivalent effects [23].

### 2.2. “Dumbbell-Shape” Dendrimers Based on N_3_P_3_

**Scheme 7:**
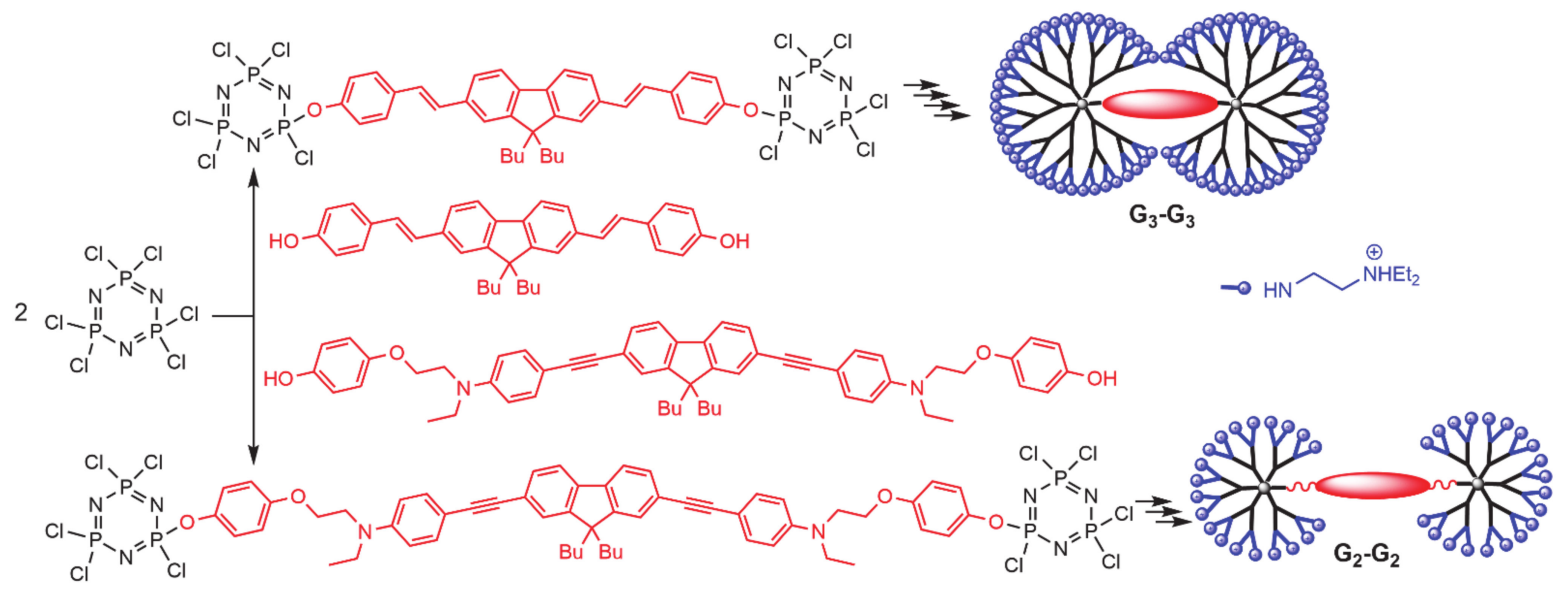
Synthesis of “dumbbell-shape” dendrimers having two AB_5_ derivatives of N_3_P_3_ linked to a fluorescent core.

Dendrimers built from two AB_5_ derivatives of N_3_P_3_, linked through a bifunctional fluorophore core, have dumbbell-shape structures, as shown, in particular, in Scheme 7. Starting from the 10 Cl of the cyclotriphosphazenes, the synthesis of the dendrimer was carried out using two repetitive steps, classically used for the synthesis of phosphorhydrazone dendrimers, i.e., the substitution reaction of Cl with hydroxybenzaldehyde, and the condensation reaction of the aldehydes with H_2_NNMeP(S)Cl_2_ [24]. The synthesis was carried out up to the second and third generations of dumbbell-shape dendrimers, and *N*,*N*-diethylethylenediamine was grafted as terminal groups in the last step, to induce solubility in water. The dendrimer built from the fluorophore having alkene bonds (blue emitter) was used for imaging in vivo the vascular network of a rat olfactory bulb [25]. The dendrimer built from the fluorophore having alkyne bonds (green emitter) was used for imaging the blood vessels of a *Xenopus laevis* tadpole [26].

**Scheme 8:**
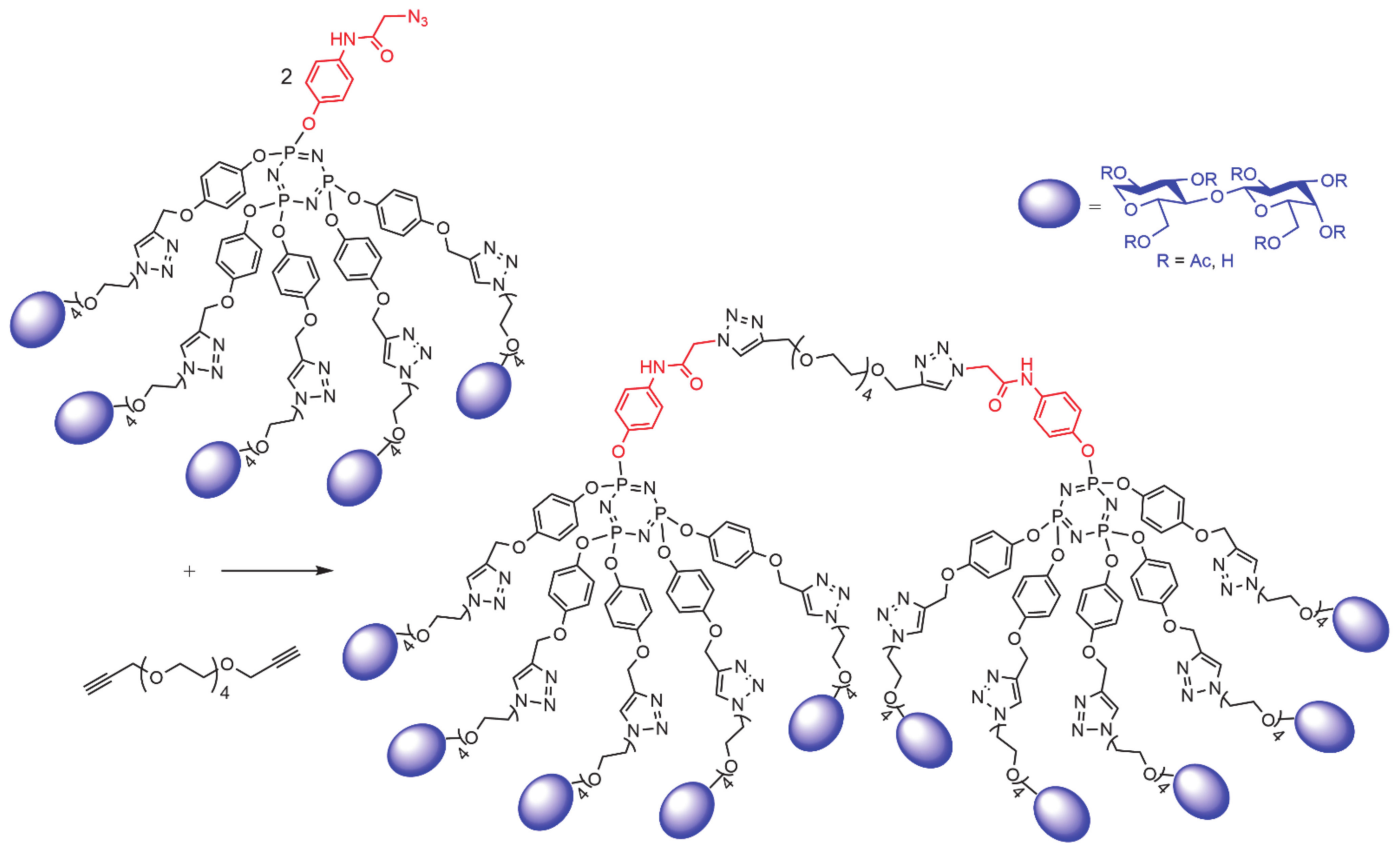
Dumbbell-shape dendrimer obtained by a double “click” reaction onto dipropargylated tetraethylene glycol.

Two equivalents of the glycosylated dendron shown in Scheme 6 were associated with a tetraethylene glycol core, functionalized with an alkyne on both sides. A double-click reaction in the presence of a copper catalyst provided another type of dumbbell-shape dendrimer (Scheme 8) [23].

### 2.3. N_3_P_3_ Dendrons for the Synthesis of Janus Dendrimers

**Scheme 9:**
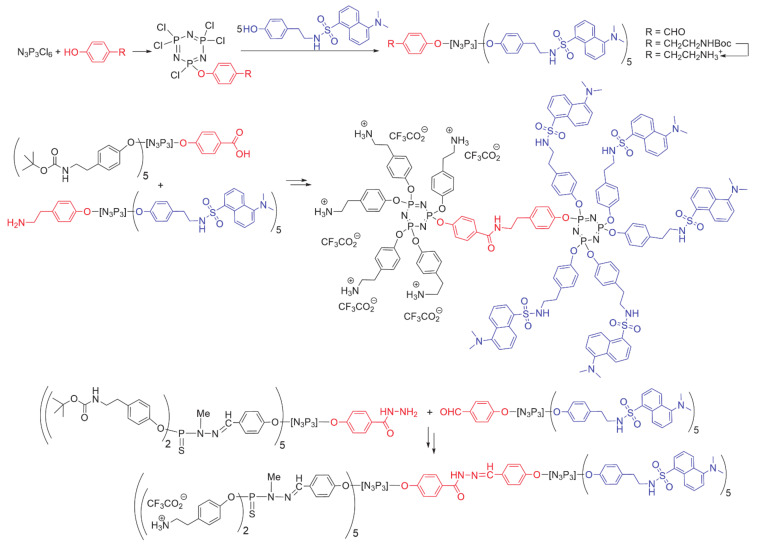
Synthesis of fluorescent dendrons, and their association with other dendrons based on N_3_P_3_ for the synthesis of two types of Janus dendrimers.

The presence of a functional group at the core of dendrons can be used for grafting to a different dendron, to afford Janus dendrimers that are dendrimers possessing two different terminal functions, located in two areas (two faces) of the surface [27]. A series of Janus dendrimers was synthesized, in which one of the faces contained fluorescent groups, and the other face, water-solubilizing groups. The first step for the synthesis of the fluorescent dendrons was the reaction of one equivalent of hydroxybenzaldehyde or one equivalent of Boc-protected tyramine with N_3_P_3_Cl_6_. In a second step, five equivalents of dansyl phenol were reacted to afford small fluorescent dendrons [28]. The second face of the Janus dendrimers was constituted by other dendrons having either a carboxylic acid or a hydrazide at the core and Boc-protected tyramine on the surface. They were synthesized by reacting first five chlorides with the Boc-protected tyramine, then one chloride to graft the carboxylate group on the cyclotriphosphazene. Two methods were used to get the Janus dendrimers. One method was the peptidic coupling of the dendron functionalized by tyramine with the dendron functionalized by a carboxylic acid. The second method was the condensation between the dendron functionalized by an aldehyde and the dendron functionalized by the hydrazide (Scheme 9) [29].

**Scheme 10:**
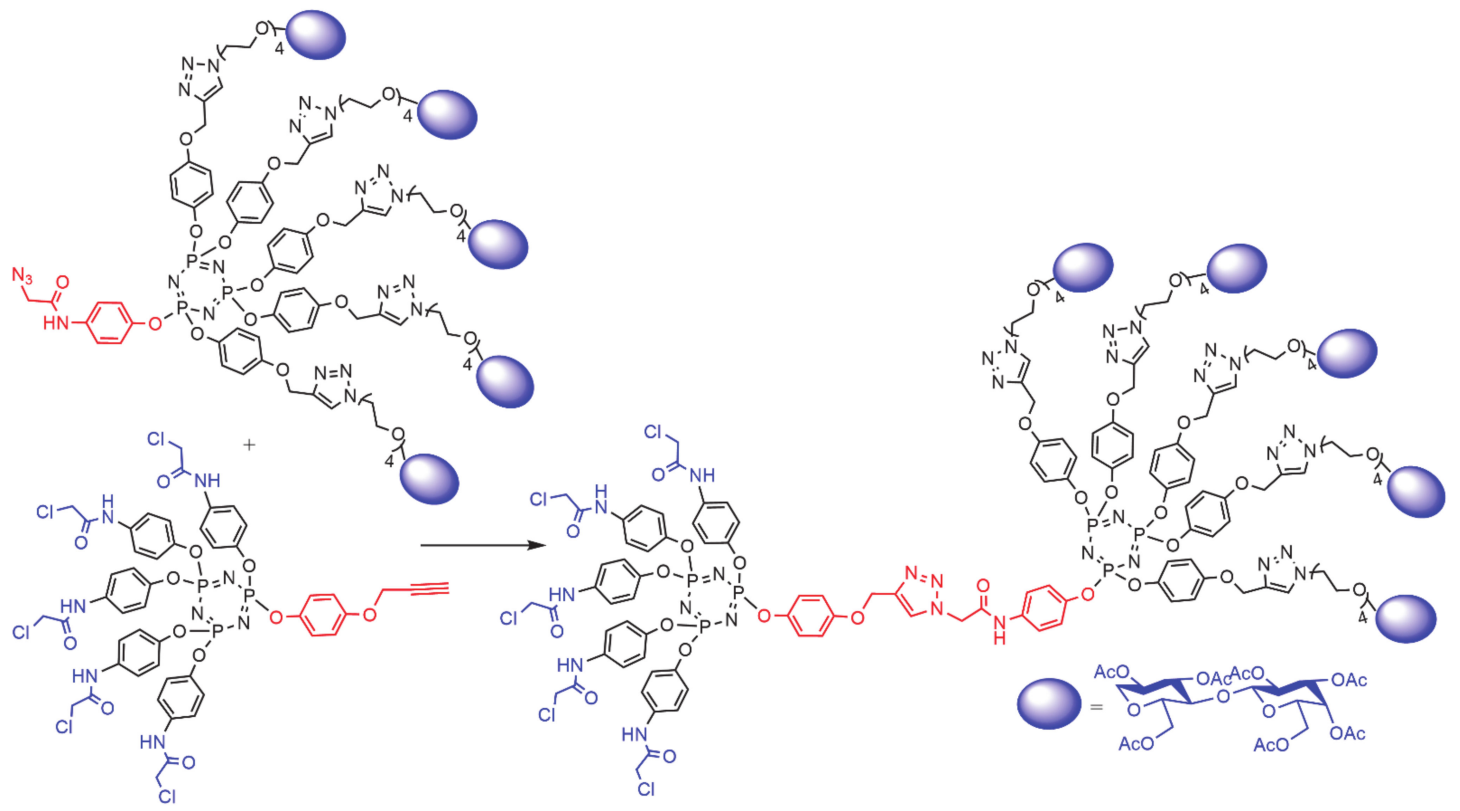
Synthesis of Janus dendrimers having two different AB_5_ derivatives of N_3_P_3_ associated via a click reaction.

Another example of Janus dendrimer was based on the dendron shown in Scheme 6 and Scheme 8, which was associated by click reaction with another small dendron bearing a propargyl group at the core and five acetyl chloride functions (Scheme 10). The presence of such functional groups on one side of this Janus dendrimer potentially offers the possibility to continue the growing of dendritic branches on one side [23].

## 3. Dendrons Based onAB_5_ Derivatives of N_3_P_3_ as Core and Their Use for the Synthesis of Nanomaterials

### 3.1. Nanomaterials Obtained by Covalent Grafting of N_3_P_3_ Dendrons to Solid Surfaces

**Figure 2:**
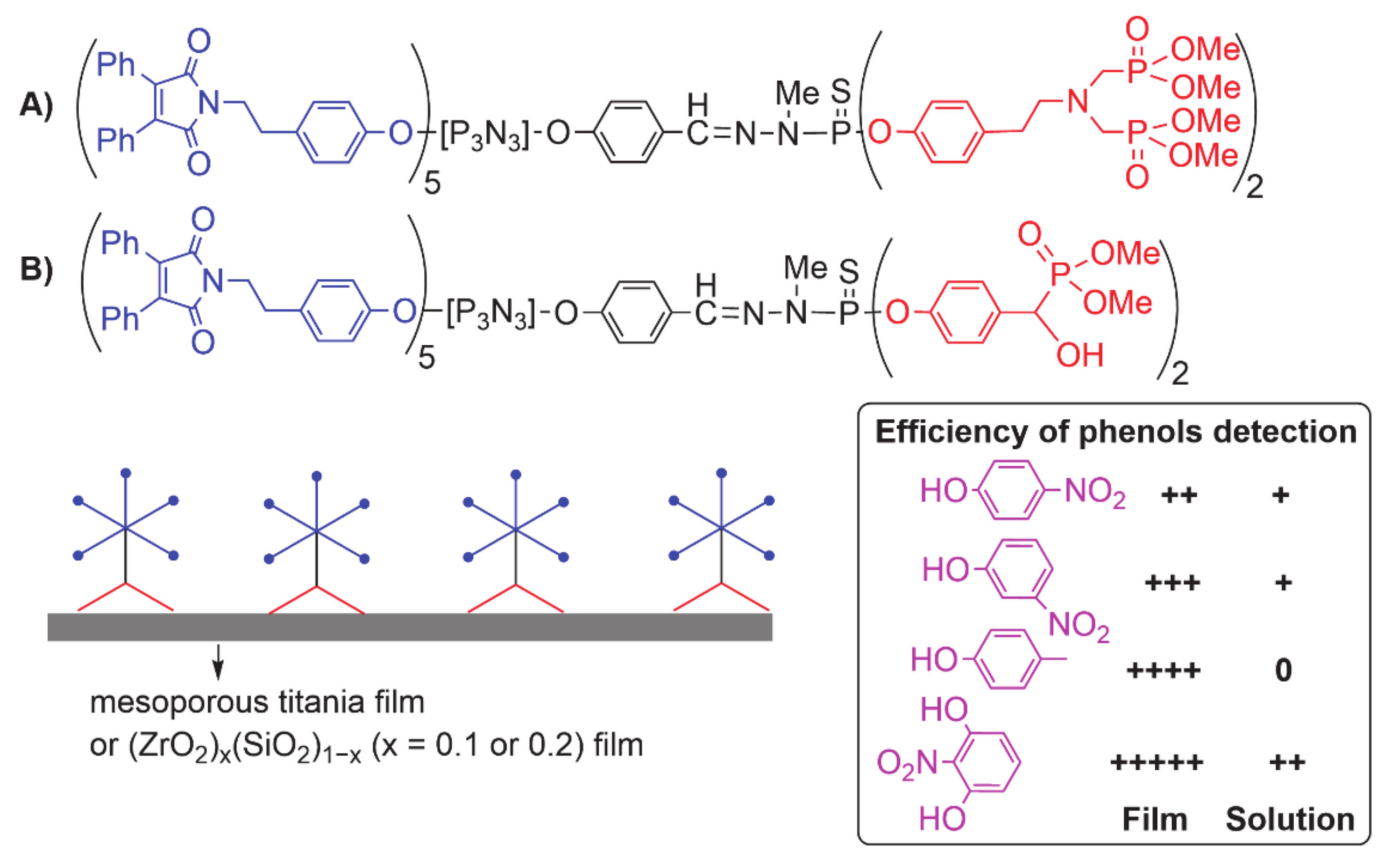
Dendrons grafted to solid surfaces. (**A**) Dendron grafted to mesoporous titania films. (**B**) Dendron grafted to (ZrO_2_)_x_(SiO_2_)_1−x_ films surface. In the frame: efficiency of the dendron shown in (**A**) on titania or in solution for the detection of nitrophenols.

Besides their utility for the synthesis of diverse types of dendrimers, as illustrated in the previous paragraphs, dendrons based on cyclotriphosphazene as core were used for the covalent modification of different hard surfaces. In the first example, two small dendrons functionalized with five maleimide fluorophores, then with either two azabisphosphonate (Figure 2A) or two α-hydroxyphosphonate groups (Figure 2B), were used for the grafting to thin films. The grafting was carried out on nanocrystalline mesoporous titania films, which became brightly fluorescent, using the first dendron. With the second dendron, the grafting was carried out on (ZrO_2_)_x_(SiO_2_)_1−x_ (x = 0.1 or 0.2) films, but the quasi-complete disappearance of the fluorescence intensity was observed, probably due to the presence of the hydroxyl groups inducing a quenching. The titania film functionalized with the dendron having two azabisphosphonate groups was used as chemical sensor, which exhibited high sensitivity to phenolic OH moieties. Different types of hazardous phenols were tested as solutions in chloroform or ethanol. A neat decrease in the fluorescence intensity of the films in the presence of phenol solutions was observed, especially with solutions containing either resorcinol or 2-nitroresorcinol. Interestingly, the quenching of fluorescence was more efficient in the solid state than in solution. This difference is probably a result of the increased spatial proximity of the fluorescent maleimide groups, which is induced by pore confinement that makes the formation of hydrogen bonds between the hydroxyl moieties of the quenchers and the carbonyl groups of the dendron easier in the solid state than in solution [30].

**Scheme 11:**
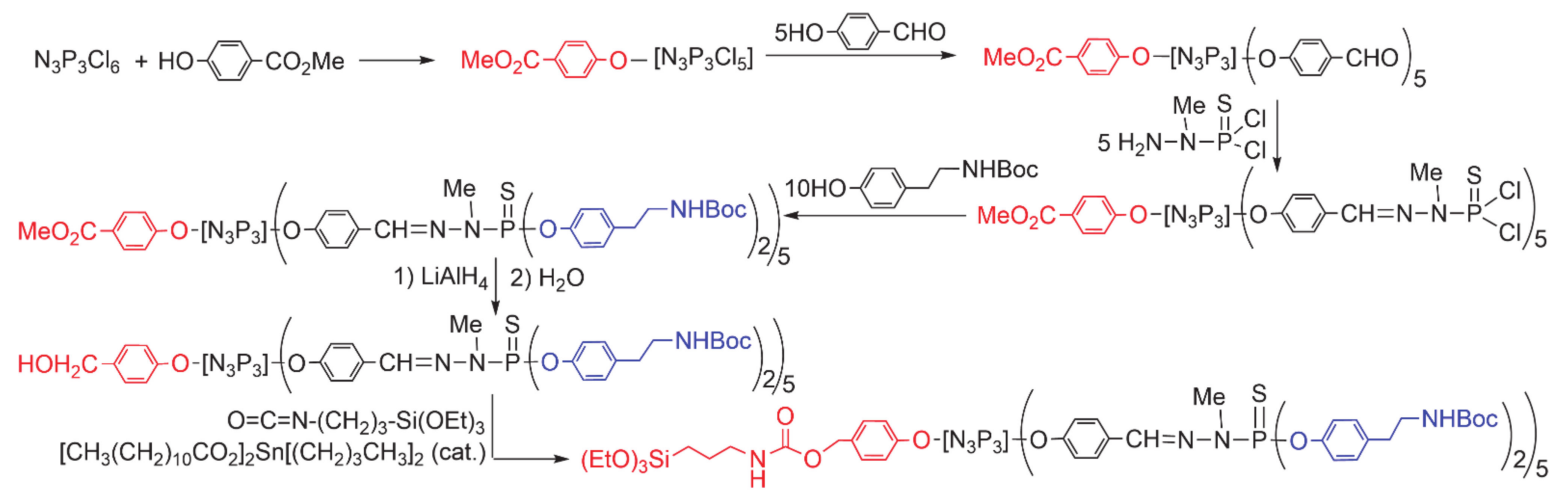
Multistep synthesis of a dendron suitable for grafting to silica, and for trapping CO_2_ after deprotection of Boc-protected tyramine.

In a second example, several dendritic structures possessing two types of functions were elaborated both to be grafted to silica (triethoxysilane) and to be able to trap carbon dioxide (primary amines). Among these structures, a dendron based on the cyclotriphosphazene functionalized with one triethoxysilane and ten primary amines was synthesized as shown in Scheme 11. The first step was the reaction of one equivalent of methyl 4-hydroxybenzoate with N_3_P_3_Cl_6_, followed by reaction with five equivalents of 4-hydroxybenzaldehyde. The growing of the dendritic branches was carried out by condensation of the aldehydes with the phosphorhydrazide H_2_NNMe-P(S)Cl_2_, followed by the reaction with Boc-Protected tyramine. The next step was the reduction and deprotection of the ester at the core, using LiAlH_4_ to generate a benzyl alcohol. The final step was the addition of the benzyl alcohol to the isocyanate OCN-(CH_2_)_3_-Si(OEt)_3_ in the presence of a stannyl catalyst [31]. This dendron was grafted to a porous silica of type SBA15 (pore diameter 6 nm). Then the Boc-protected amines were deprotected with trifluoroacetic acid (10%) in dichloromethane, followed by several washings. Measurements of CO_2_ trapping showed that this compound was active, but not the most active of the series, probably because it penetrated too far inside the pores of silica due to its small size [32].

**Figure 3:**
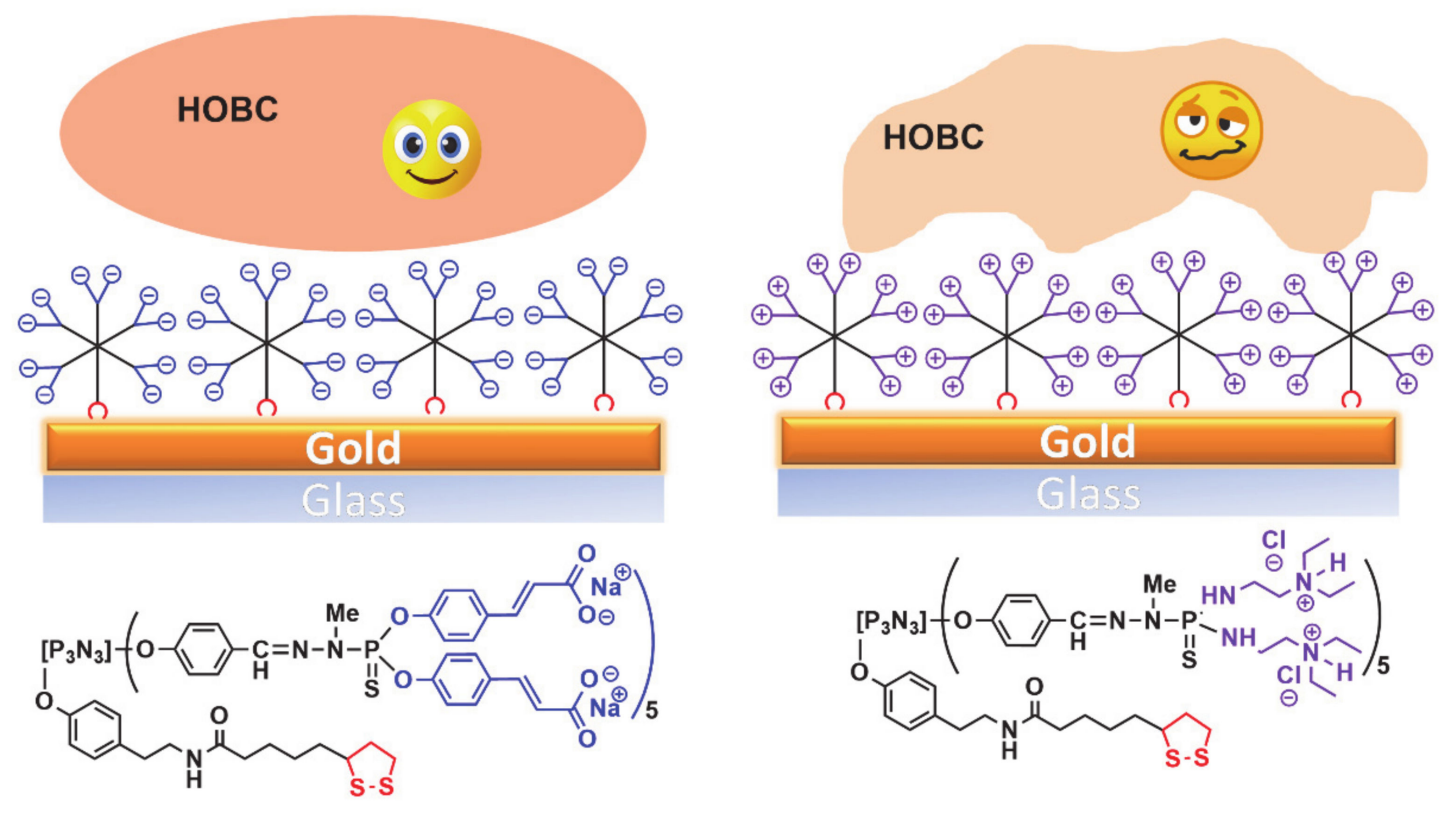
Dendrons functionalized with thioctic acid at the core for grafting to gold surfaces, and with positively or negatively charged terminal functions. The modified surfaces were used as supports for cultures of human osteoblast cells (HOBC).

In a third example, two different families of bifunctional water-soluble dendrons were synthesized. Both possess a single thioctic acid at the core (grafted in the first step of the synthesis), suitable for grafting to a thin gold layer coated on a glass surface. Generations 1 and 2 were synthesized and functionalized by either ammoniums or carboxylates as terminal functions. Only the first generations could be grafted to the gold surface, the thioctic acid being too buried inside the dendritic structure of second generation to be accessible for grafting to the gold surface. The surfaces modified by the positively or negatively charged dendrons were then used for studying their interaction with cells, in particular for the culture of human osteoblast cells (HOBC). It was shown that polycationic dendrons provoked cell apoptosis, whereas negatively charged dendrons supported cell adhesion and proliferation, as illustrated in Figure 3 [33].

### 3.2. Non-Covalent Grafting of N_3_P_3_ Dendrons to Graphene Coated Co Nanoparticles

**Scheme 12:**
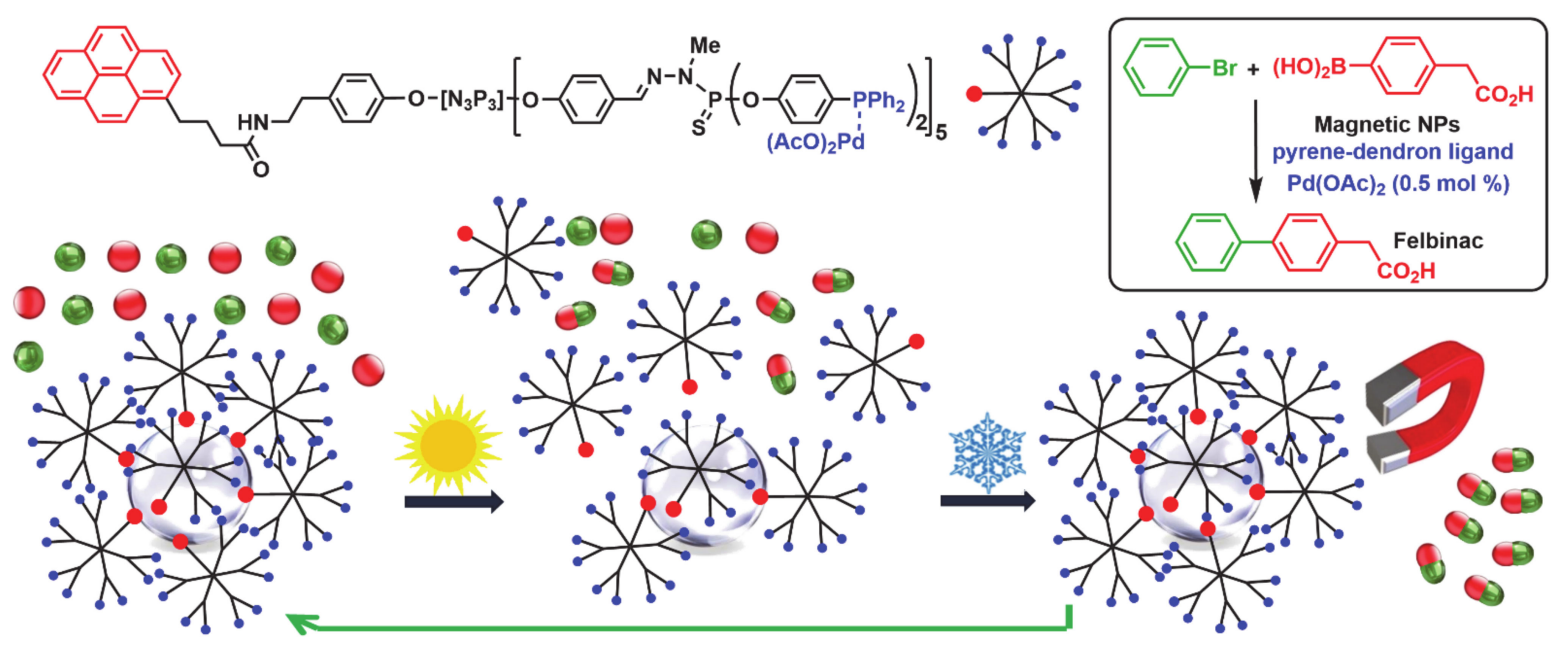
Non-covalent grafting of catalytic dendrons onto cobalt nanoparticles covered by graphene layers, and their use as catalysts in Suzuki couplings. Example of coupling for the synthesis of Felbinac. Recovering and re-use were carried out eleven times, with a quantitative yield in Felbinac in all cases.

Besides the covalent grafting of dendrons to surfaces shown above, a few examples concern the non-covalent (and thus reversible) π-stacking of dendrons functionalized with a pyrene at the core on graphene surfaces encapsulating cobalt nanoparticles (Co-NPs). In the first example, the pyrene was grafted in the first step, and the surface of the dendron was functionalized in the last step with triphenyl phosphine derivatives, as suitable ligands for the complexation of palladium. At room temperature, in the presences of cobalt nanoparticles coated with a few graphene layers, the dendrons were associated to Co-NPs by π-stacking. When heating, the dendrons were dissociated from the Co NPs, and performed catalysis in homogeneous conditions. Upon cooling, the dendrons associated back to the Co NPs. Co nanoparticles are magnetic, thus the Co-NPs covered by the dendrons could be easily recovered using a magnet, and re-used. This concept is illustrated in Scheme 12, and was applied for catalyzing Suzuki couplings. It was applied in particular to the synthesis of Felbinac, which is a nonsteroidal anti-inflammatory drug. The first catalytic run gave Felbinac in quantitative yield. The catalyst and the product were cleanly and easily separated using a neodymium magnet to recover the catalytic Co-NPs covered by graphene and the dendrons. The recovered catalyst was used in a new catalytic run, in which Felbinac was also isolated quantitatively. The catalytic Co-NPs covered by the dendrons were recovered and reused 11 times, and afforded Felbinac with the same efficiency (quantitative yield) at the 12th run than at the first run (Scheme 12) [34].

**Scheme 13:**
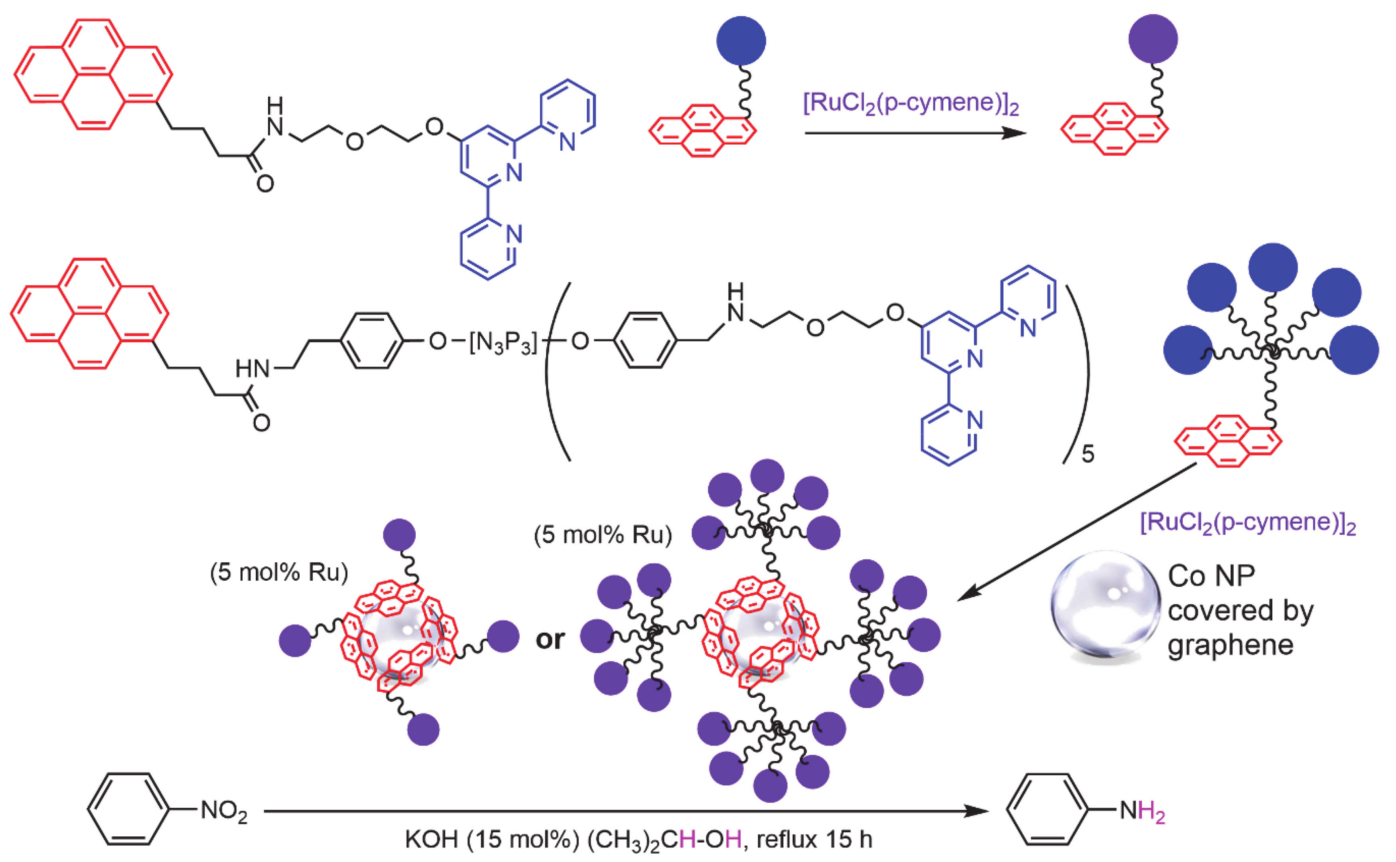
Another example of non-covalent grafting of catalytic dendrons onto cobalt nanoparticles covered by graphene layers. The lower part of the Scheme displays the catalyzed nitroarene transfer hydrogenation from 2-propanol.

A recent catalytic-related example concerned a pyrene monomer and a small pyrene dendron (pyrene grafted in the first step), both equipped with the terpyridine ligand, suitable for the complexation of ruthenium complexes. The monomer and the dendron were non-covalently grafted as previously on the surface of cobalt NPs covered with graphene (Scheme 13). Both were used in catalyzed nitroarene transfer hydrogenation from 2-propanol. Recycling and recovery of the catalysts-Co NP with a magnet could be carried out seven times with still 100% yield in the eighth experiment with the dendritic catalyst, but not with the monomeric catalyst, which could be efficiently reused only four times [35].

**Figure 4:**
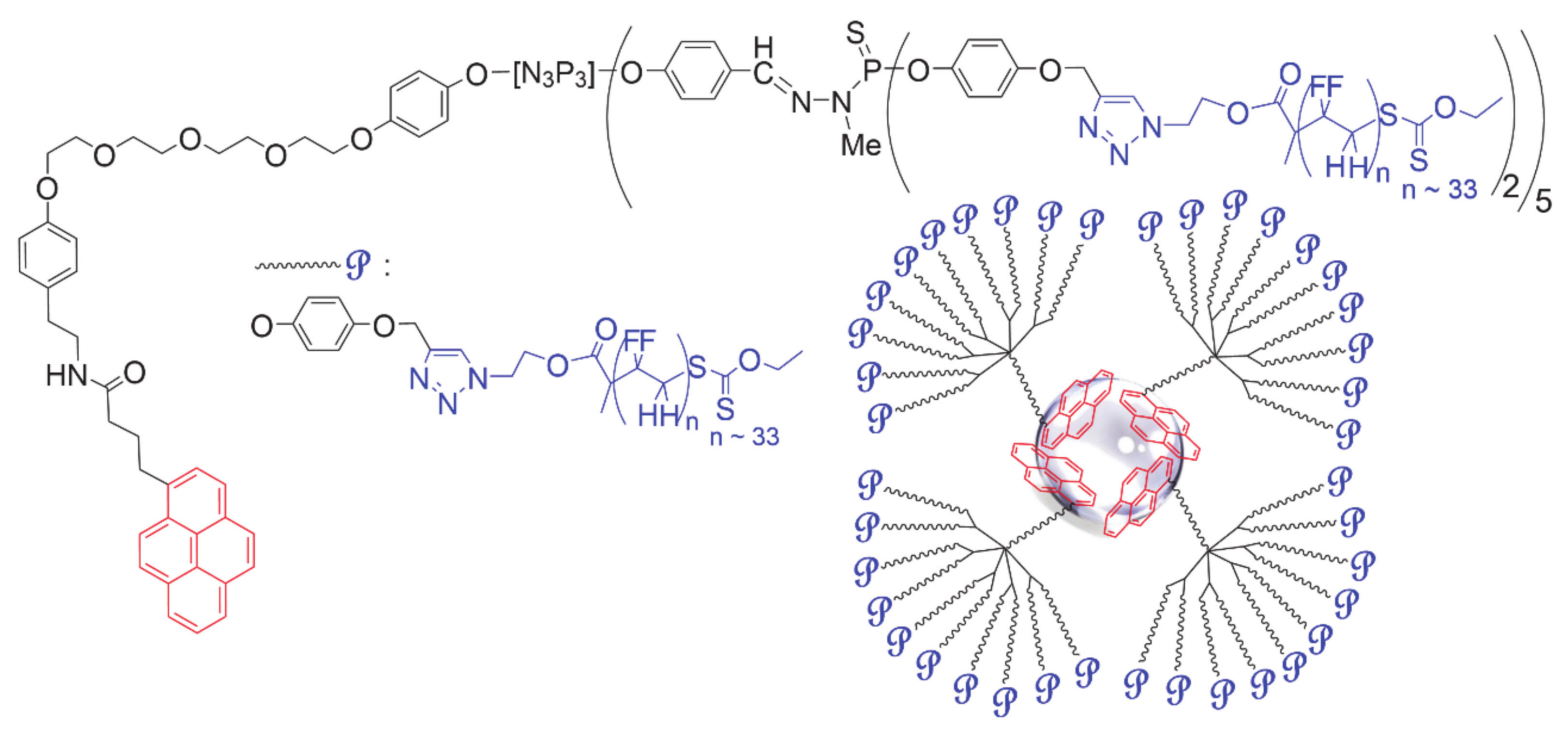
Chemical structure of a dendron bearing vinylidene fluoride polymers on the surface, and used for coating cobalt nanoparticles covered by graphene.

In another example using the pyrene-graphene association, the link between the dendron and the pyrene was longer than in the previous case. In this case, five hydroxybenzaldehydes were first grafted to the cyclotriphosphazene, prior to the pyrene derivative. The surface of the dendron was functionalized with poly(vinylidene fluoride) (PVDF) polymers, using a Huisgen [3 + 2] cycloaddition (“click chemistry”). PVDF is an interesting fluoropolymer, constituted of CF_2_-CH_2_ units. It has remarkable properties such as thermal stability, chemical inertness to solvents and acids as well as piezo-, pyro- and ferroelectric properties [36]. The dendron shown in Figure 4 was used to interact by π-stacking with the graphene surface of cobalt NPs covered by graphene layers. A thermo-responsive behavior was displayed, showing that the interaction is reversible when heating, in accordance with the previous cases shown in Scheme 12 and Scheme 13 [37].

## 4. Fluorescent Dendrons Based on AB_5_ Derivatives of N_3_P_3_ as Core, Used in Biology

Diversely functionalized phosphorus dendrimers have many biological properties against cancers, chronic inflammations (rheumatoid arthritis, encephalomyelitis, psoriasis, etc.), tuberculosis, neurodegenerative diseases, and as carriers of biomolecules or drugs [38]. In order to decipher their mechanisms of action, it is often necessary to follow their behavior inside cells and in the body. To do that, a fluorescent group has been attached in many cases to the core of the dendrimer, thus providing a fluorescent dendron. The following paragraphs will be organized depending on the nature of the fluorophore linked to the cyclotriphosphazene core.

### 4.1. 2,3-Diphenyl Maleic Tyramine Derivative at the Core of N_3_P_3_ Dendrons

**Figure 5:**
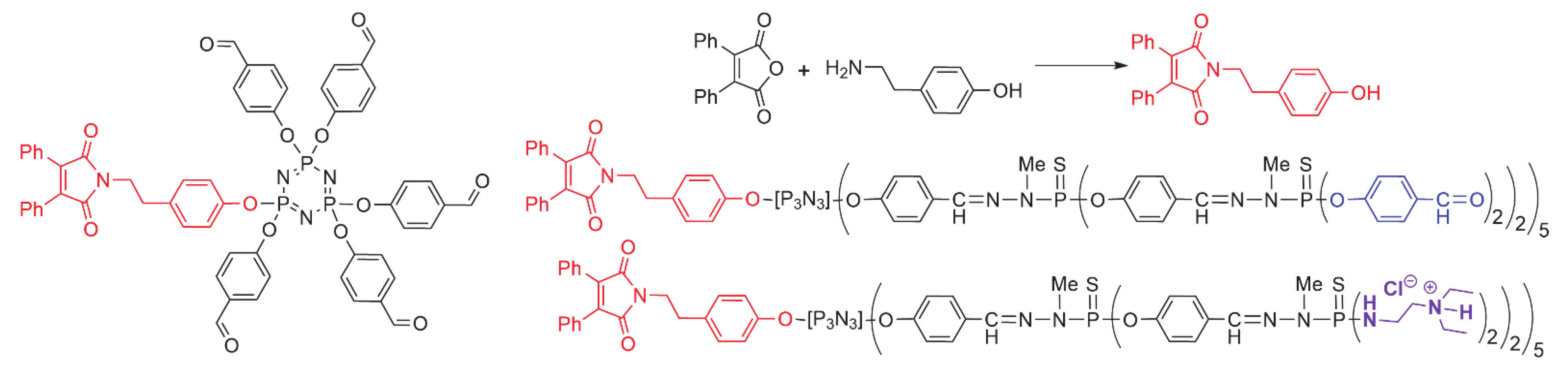
Dendrons functionalized with a single fluorescent maleimide at the core and with either 20 aldehyde or 20 ammonium terminal functions.

A fluorescent maleimide derivative was synthesized by reaction of 2,3-diphenyl maleic anhydride with tyramine. Five equivalents of this fluorophore linked to N_3_P_3_ afforded the chemical sensor shown in Figure 2. The same fluorophore was grafted as a single function at the core of dendrons, functionalized in the first step by five equivalents of hydroxybenzaldehyde. These dendrons were built up to the second generation (20 aldehyde terminal functions, Figure 5). These compounds were first synthesized with the aim of determining the influence of the burying of the fluorophore inside the dendron on the fluorescence properties. The fluorescence quantum yield measured in dichloromethane decreased from 77 for the generation zero (5 aldehydes), to 22 for the first generation, and 20 for the second generation. Such result demonstrated a negative influence of the encapsulation of the maleimide fluorophore inside the dendritic structure [39]. The same family of dendrons was functionalized with ammonium terminal functions, instead of aldehydes, to afford water-soluble compounds. The fluorescence of the second generation (Figure 5) was largely decreased in water compared to dichloromethane. The cytotoxicity of this dendron was assessed toward two cancerous cell lines, and was found relatively low. This dendron was synthesized with the aim of monitoring transfection experiments, thanks to the presence of ammonium groups [40]. It was demonstrated that the dendron was able to interact with plasmid DNA (BACE-GFP), but the fluorescence was too low to be used to monitor the transfection [41].

**Figure 6:**
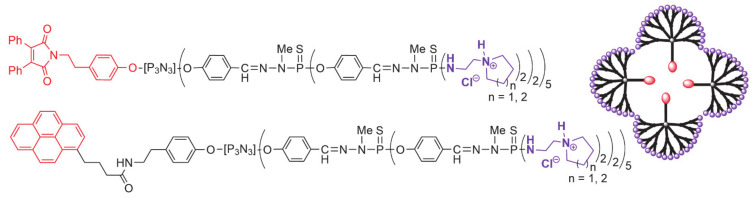
Amphiphilic dendrons functionalized with either maleimide or pyrene at the core and 20 cyclic ammoniums on the surface (2-(pyrrolidin-1-yl)ethan-1-amine (five-membered ring) or 2-(piperidin-1-yl)ethan-1-amine (six-membered ring). Schematized association of these dendrons in micelle.

The same family of dendrons, generations 1 and 2, was functionalized with cyclic ammoniums instead of linear ammoniums. The cyclic ammoniums were salts of 2-(pyrrolidin-1-yl)ethan-1-amine and of 2-(piperidin-1-yl)ethan-1-amine. Besides maleimide, a pyrene derivative (the one already shown in Scheme 12) was also used as fluorescent and hydrophobic core (Figure 6). In both cases, the synthesis started with the grafting of five equivalents of hydroxybenzaldehyde to the N_3_P_3_ core. The ability of these amphiphilic dendrons to form micelles in water was assessed. Whatever the generation and the type of cyclic ammoniums, the dendrons built from the pyrene derivative at the core had a Critical Micelle Concentration (CMC) between 1 and 4 μM. The maleimide series less easily formed micelles, and displayed large differences in the CMC, depending on the generation of the dendron. The CMC was 76.9 and 73.3 μM for the first generation, 35.3 and 26.3 μM for the second generation, in both cases having pyrrolidinium and piperidinium terminal functions, respectively. These dendron-based micelles were tested against a panel of tumor cell lines. The most active compound against all cell lines was the first generation dendron having the pyrene at the core and 10 piperidiniums on the surface. This compound displayed IC_50_ values (minimum concentration necessary to kill 50% of the cells) of 0.28 μM (HL60), 0.36 μM (K562), and 1.07 μM (HCT116). These values are slightly below the CMC for this compound [42].

**Figure 7:**
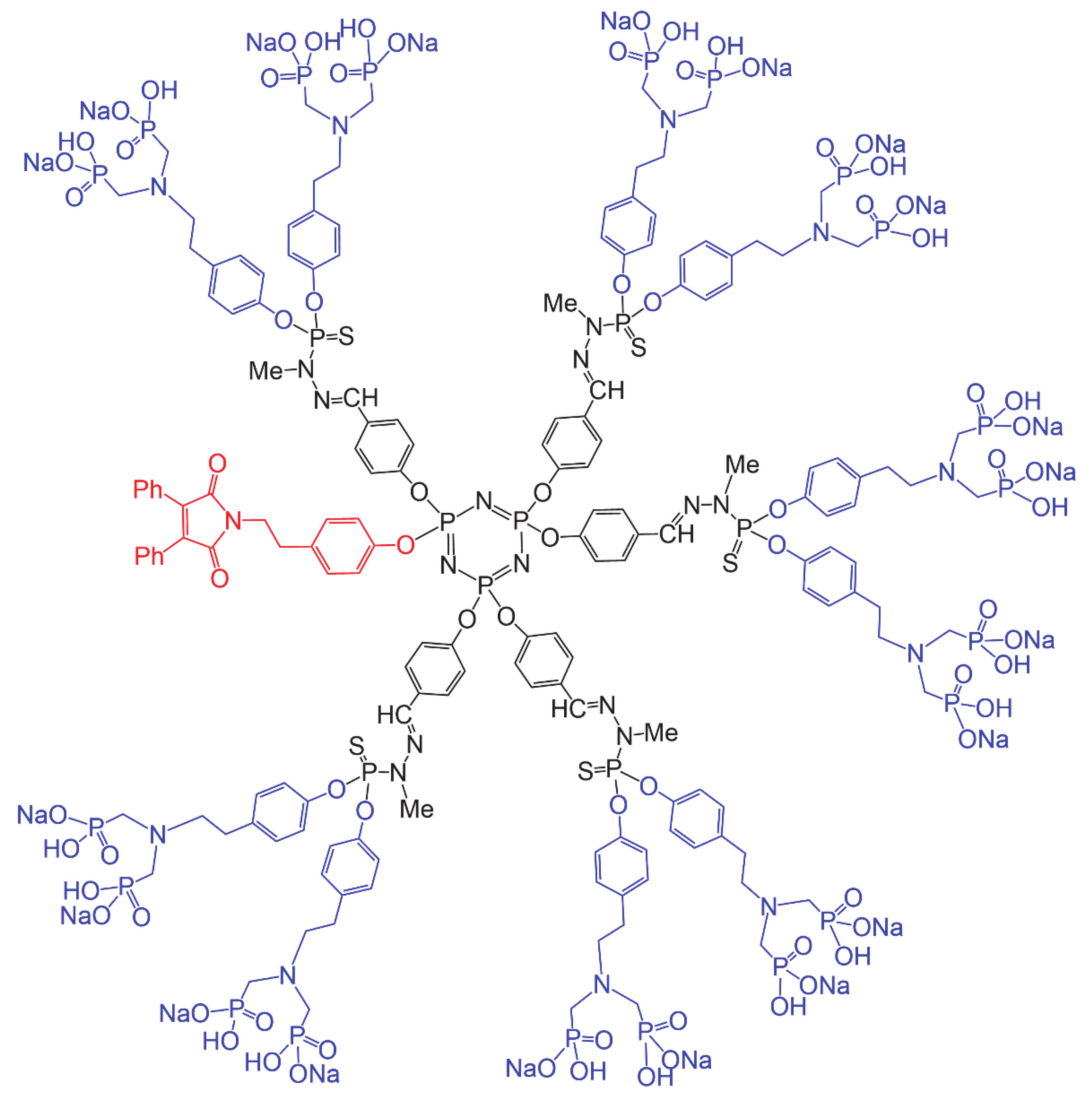
Full chemical structure of a first generation dendron having a maleimide at the core and 10 azabisphosphonate terminal functions.

A first-generation dendrimer having as terminal functions 12 azabisphosphonate groups based on tyramine was shown to be able to activate monocytes, which are a key cell population of the human innate immunity. In order to try to decipher the mechanisms and consequences of this activation, an analogous fluorescent dendron having the maleimide at the core and 10 azabisphosphonate terminal functions (Figure 7) was synthesized. The maleimide derivative was grafted in the very first step of the synthesis. It was used for FRET experiments (fluorescence resonance energy transfer) with phycoerythrin-coupled antibodies associated to different receptors, to determine the eventual involvement of a specific receptor [43].

The same family of dendrimers/dendrons was shown to be able to multiply, frequently by several hundred folds, the number of natural killer (NK) cells, which are another key cell population of the human innate immunity, able to fight against viral and bacterial infections, and against many cancers. Several generations of dendrimers bearing diverse negatively charged terminal functions were also synthesized, but the most active compound remained the first generation functionalized with azabisphosphonate functions [44]. It was shown later on that both the plain dendrimer (12 azabisphosphonate terminal functions) and the fluorescent dendron shown in Figure 7 (10 azabisphosphonate terminal functions) added to ex vivo cultures of peripheral blood mononuclear cells from healthy volunteers or from cancer patients with multiple myeloma enabled the efficient proliferation of NK cells, which were proved to exhibit in vivo anti-cancer activity [45].

### 4.2. Julolidine at the Core of N_3_P_3_ Dendrons

**Figure 8:**
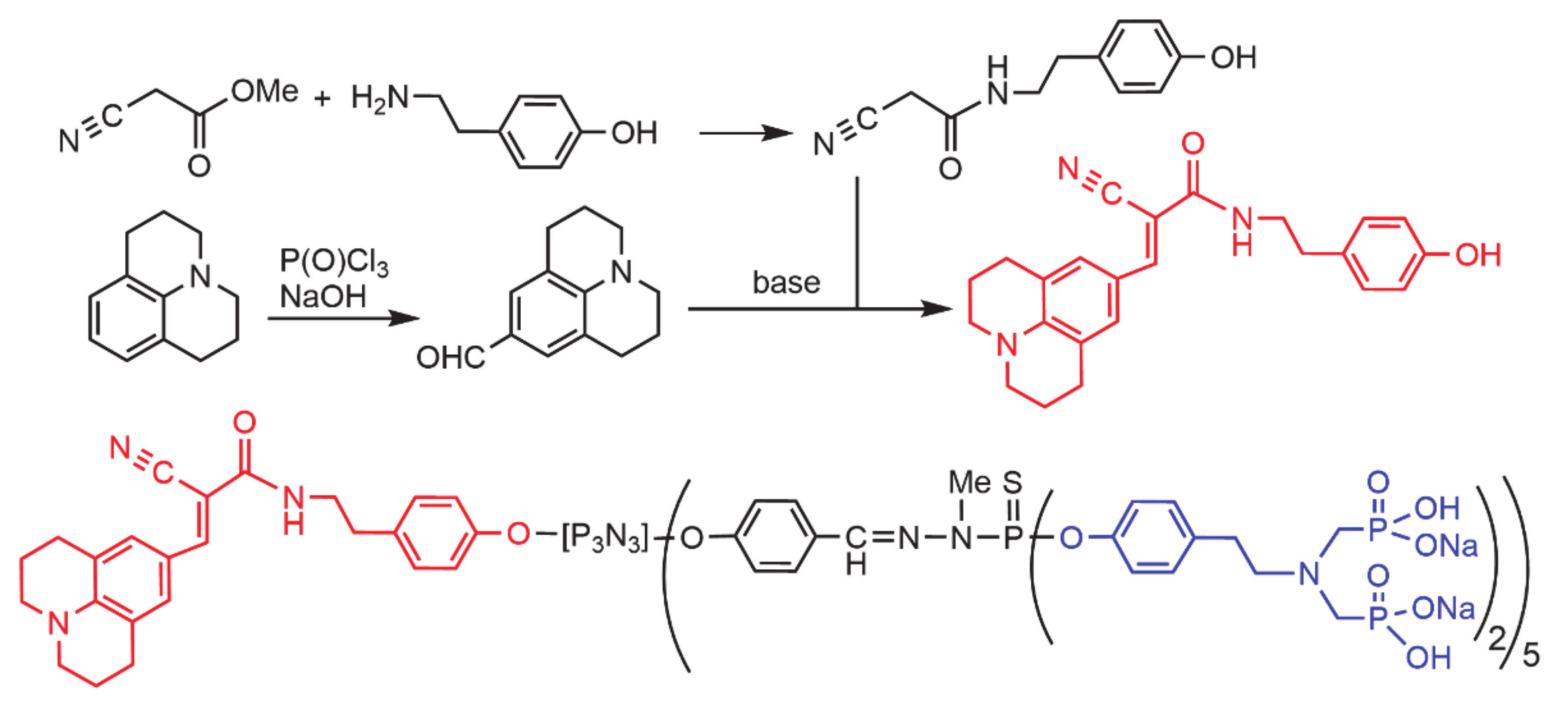
Synthesis of a julolidine derivative, and its use for the functionalization of an azabisphosphonate dendron.

In view of all the properties of the dendrimers ended by azabisphosphonate terminal functions, a structure/activity relationship was carried out, together with the desire to decipher their complex interactions with biological processes. Indeed, it was demonstrated that this family of dendritic compounds offers an alternative strategy to the treatment of chronic inflammatory diseases such as rheumatoid arthritis [46] and multiple sclerosis [47]. Besides maleimide, other fluorescent groups were linked to the core of the dendrons, in particular based on julolidine to help in deciphering the biological mechanism of action. Indeed, the tracking of cellular or molecular events often requires the use of fluorescent labels. In this regard, having in hand several fluorescent analogs of a given compound with various λ_em_/λ_ex_ is somehow convenient to design biological experiments. The suitably functionalized julolidine was synthesized first by a Vilsmeyer formylation on commercially available julolidine, to obtain 4-formyljulolidine in quantitative yield. In parallel, the reaction of cyanomethyl acetate with tyramine afforded quantitatively 1-cyano-N-acyl tyramine. Coupling of both compounds by Knoevenagel condensation proceeded in high yield to get the desired julolidine derivative equipped with a phenol (Figure 8). This compound was used for grafting to the single remaining chloride of cyclotriphosphazene equipped with five benzaldehyde functions. The growing of the branches and the grafting of azabisphosphonate functions afforded the desired fluorescent dendron. This dendron was also efficient for inducing the activation of monocytes [48].

This fluorescent julolidine dendron was used to investigate the immunomodulatory effects of these dendritic azabisphosphonate derivatives. It was shown that they inhibited the activation, and therefore the proliferation, of CD4^+^ T cells in cultures with IL-2 (interleukine) without affecting their viability. The presence of a larger quantity of IL-2 available induced the rapid enrichment of NK cells. The fluorescent dendron allowed us to demonstrate a direct interaction with the T cells [49].

**Figure 9:**
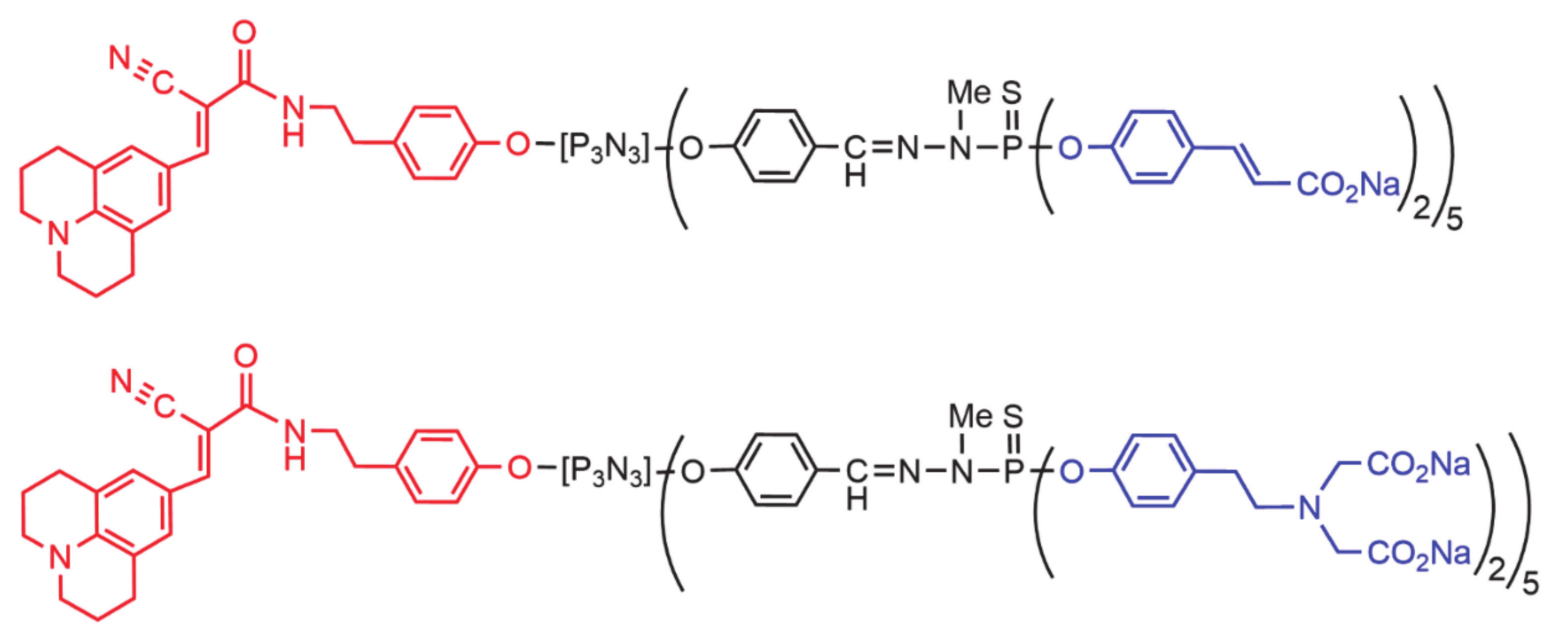
Examples of dendrons functionalized by one julolidine derivative at the core and 10 carboxylate or azabiscarboxylate terminal functions.

Besides azabisphosphonate groups, other negatively charged functions were also tested, in particular two types of carboxylates. The efficiency of these compounds was compared with that of the dendron shown in Figure 8. Dendrimers functionalized with trans-cinnamic acid were first used for the elaboration of nanostructured materials [50]. The plain dendrimer and the corresponding fluorescent dendron (Figure 9) were tested on membrane models (monolayers and multi-lamellar vesicles), and on monocytes. It was shown that the cinnamate dendrimer and dendron strongly disturb and alter the structure and the properties of model membranes, and they were found toxic towards monocytes. A totally different behavior was observed with the azabisphosphonate dendrimer and dendron, which had a low impact on the model membranes, and no toxicity towards monocytes [51].

The exact equivalent of the azabisphosphonate terminal functions was also synthesized in the carboxylate series, i.e., the azabiscarboxylate dendrimer [52]. The corresponding fluorescent julolidine dendron was also synthesized (Figure 9). Interaction of these macromolecules with monocytes revealed that the fluorescent derivative having azabisphosphonate terminal functions can bind both non-specifically and specifically to the membrane of human monocytes. The specific binding led to the internalization of the phosphonate derivative by human monocytes. In contrast, the corresponding carboxylate derivative interacted only non-specifically with human monocytes, and was not internalized [53].

Besides changing the surface terminal functions, the internal structure of the dendrimer was also modified. A large number of dendrimers functionalized in all cases with azabisphosphonate terminal functions were synthesized starting from different types of scaffolds, and tested for the activation of monocytes. Purely organic dendrimers (PAMAM, PPI and *p*-Lys) had no activity, whereas partly inorganic dendrimers, i.e., dendrimers bearing phosphorus or silicon at the branching points, were all active. The importance of the internal structure on the properties was clearly illustrated for the first time. The key feature was the “directionality” of the macromolecules, i.e., the repartition of the terminal functions as shown in the 3D modeling by molecular dynamics. All the active dendrimers have the terminal functions localized in only a part of the structure, whereas all the non-active dendrimers have their terminal functions spread all over the surface [54]. In continuation of this work, dendritic structures having a lower number of azabisphosphonate groups (10, 8, 4, 2) or a higher number (16, obtained from a cyclotetraphosphazene core) were also synthesized and tested for their efficacy to control arthritis in a mouse model. Only compounds having 10 (fluorescent dendron) or 12 azabisphosphonate terminal functions were highly active. All-atom molecular dynamics simulations of all these dendritic structures in explicit solvent demonstrated also that the most directional compounds are the most active [55].

### 4.3. A Near Infra-Red (NIR) Fluorophore at the Core of N_3_P_3_ Dendrons

**Figure 10:**
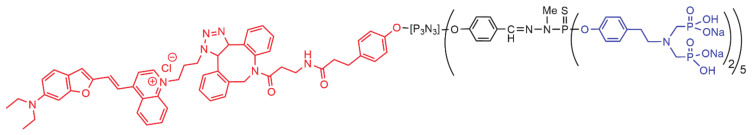
Dendron functionalized with a NIR fluorophore at the core and 10 azabisphosphonate derivatives on the surface.

On the way to clinical translation of this family of dendritic compounds, the biodistribution and the safety have to be assessed. In order to expand the types of tools to perform such experiments, a near Infra-red (NIR) fluorophore was synthesized and grafted to the core of the dendron bearing 10 azabisphosphonate terminal functions (Figure 10). The biodistribution in mice was first assessed with the julolidine dendron, then with the NIR dendron. It was shown that the julolidine dendron injected intravenously in the mouse tail can be detected in draining lymph nodes and in the liver, and in very little proportion in the kidneys at day one. At day three, it was detected in the spleen, but mainly in the liver, where it can be found until day 15. To get better insight in the biodistribution, a NIR fluorophore was designed. Indeed, the julolidine derivative is brightly fluorescent in the green, but the autofluorescence of the tissues induces a green background which precludes a good quantification of the presence of the dendron. The dendron bearing the NIR fluorophore was mainly found in the lungs and in the liver, whereas lower quantities were detected in the lung and in the kidney. Fluorescence gradually decreased with time, but could be detected up to 56 days post injection [56].

With the aim of broadening the scope of the anti-inflammatory properties of the azabisphosphonate dendritic compounds, their therapeutic efficacy in a preclinical mouse model of psoriasis induced by imiquimod was assessed. A moderate therapeutic effect was observed. The NIR dendron (Figure 10) was used for determining the skin permeation capability [57].

## 5. Other Dendrons Based on AB_5_ Derivatives of N_3_P_3_ as Core, and Used in Biology

**Figure 11:**
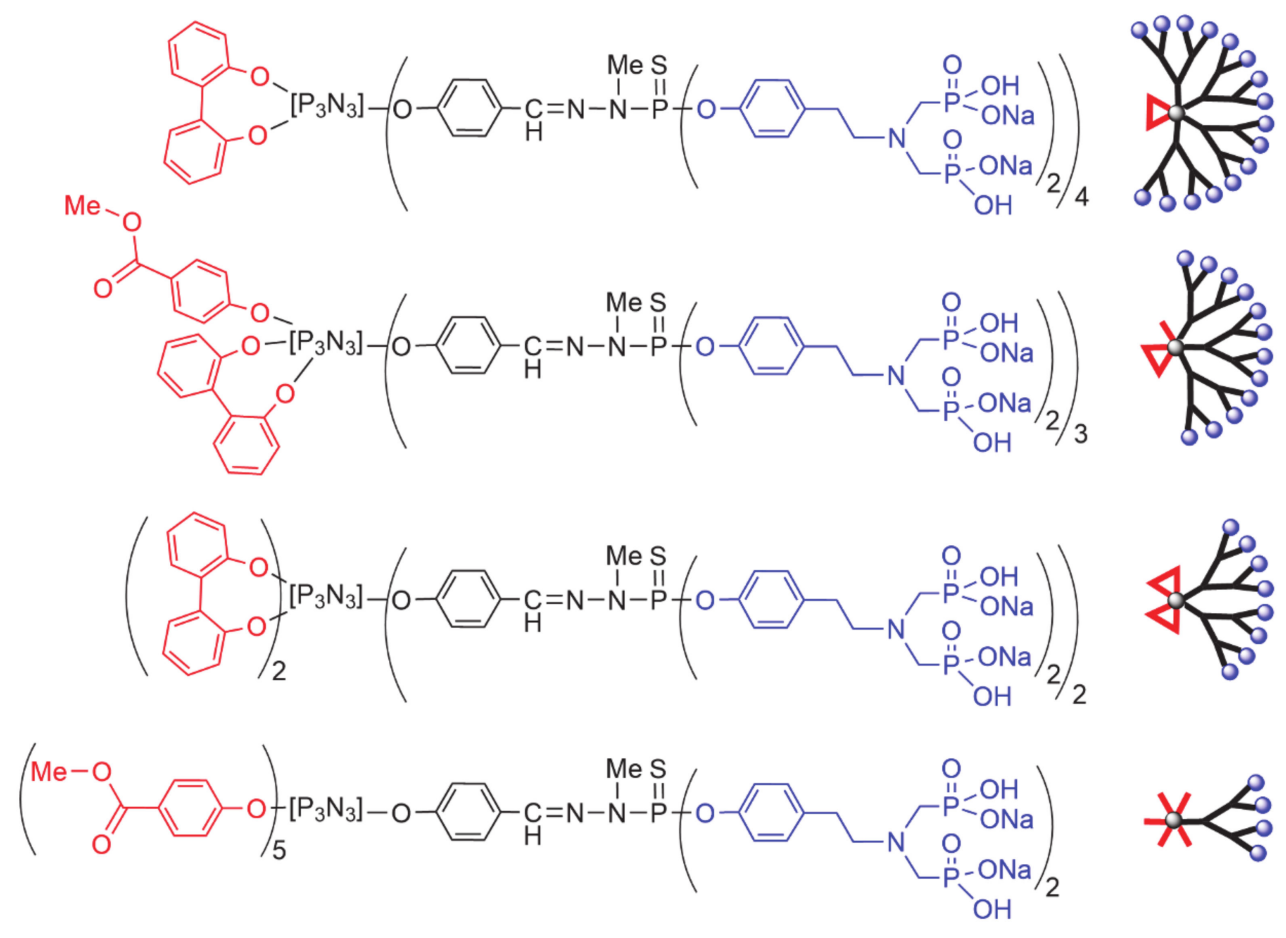
Chemical structure and schematization of dendritic structures having a decreased number of azabisphosphonate terminal functions.

The family of dendritic structures having azabisphosphonate terminal functions has been expanded by decreasing the number of terminal active functions, in order to determine the influence of surface loading on the biological properties. This goal was attained by playing with the specific functionalization of cyclotriphosphazene. The case of five reactive functions and one non-active has been exemplified in several previous Figures with a fluorophore being the non-reactive function (Figure 7, Figure 8, Figure 9 and Figure 10). Two non-active functions were obtained by reacting first 2,2′-dihydroxybiphenyl on N_3_P_3_Cl_6_. The reaction occurred on a single phosphorus atom, leading to a seven-membered heterocycle. The growing of the dendritic branches was carried out starting from the four remaining chlorides. Three non-active functions were obtained by reacting first one equivalent of 4-hydroxymethylbenzoate, then one equivalent of 2,2′-dihydroxybiphenyl on N_3_P_3_Cl_6_, affording AB_2_C_3_ derivatives. Four non-active functions were obtained by reacting two equivalents of 2,2′-dihydroxybiphenyl on N_3_P_3_Cl_6_. Finally, five non-active functions were obtained by reacting five equivalents of 4-hydroxymethylbenzoate on N_3_P_3_Cl_6_. In all these cases, the growing of the remaining branches was carried out up to obtaining azabisphosphonate terminal functions (Figure 11) [48].

Screening the bioactivity of all these dendritic structures towards human monocytes demonstrated that the compound built from the cyclotriphosphazene first functionalized with one equivalent of 2,2′-dihydroxybiphenyl, thus having eight azabisphosphonate terminal functions was still very active. However, all the other dendritic derivatives displayed a largely decreased activity, pointing out the necessity to have at least eight azabisphosphonate terminal functions [48]. Recently the same series of dendritic structures was screened for the multiplication of NK cells. It was shown that the activation of monocytes is necessary in the framework of a multistep cross-talk between monocytes and NK cells. Thus, compounds that are not able to activate monocytes are not able to multiply NK cells [45].

**Figure 12:**
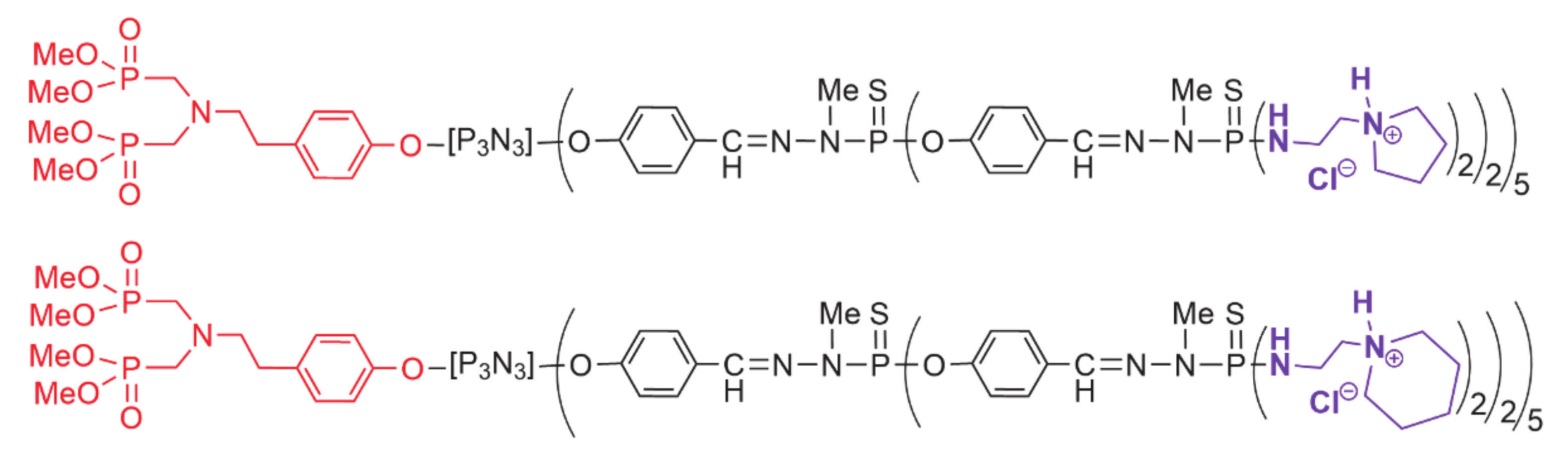
Structure of two dendrons having anti-cancer properties.

Besides the fluorescent dendrons shown in Figure 6, dendrons functionalized with the same cyclic ammoniums on the surface and bearing one azabisphosphonate group at the core instead of the fluorophore were also synthesized (Figure 12). As previously, the first step was the reaction of five equivalents of hydroxybenzaldehyde on N_3_P_3_Cl_6_, followed by the grafting of one azabisphosphonate group. The azabisphosphonate groups are less hydrophobic than the fluorescent groups, thus the CMC values were higher, especially for the first generations (153.7 and 109 μM, for pyrrolidinium and piperidinium terminal groups, respectively), but also for the second generations (60 and 45.5 μM). As already observed with the fluorescent derivatives, dendrons containing piperidinium terminal groups displayed higher cytotoxicity than the corresponding dendrons containing pyrrolidinium terminal groups, and the second generation was more cytotoxic than the first. The second generation bearing the six-member ring ammonium was found to be much safer than free DOX when it treated A549, PC3, HCT116, and MDA-MB-231 tumor cell lines, as it displayed cell selectivity. Indeed, this dendrimer had an adequate safety profile versus normal mouse fibroblast L929 cells [42].

**Figure 13:**
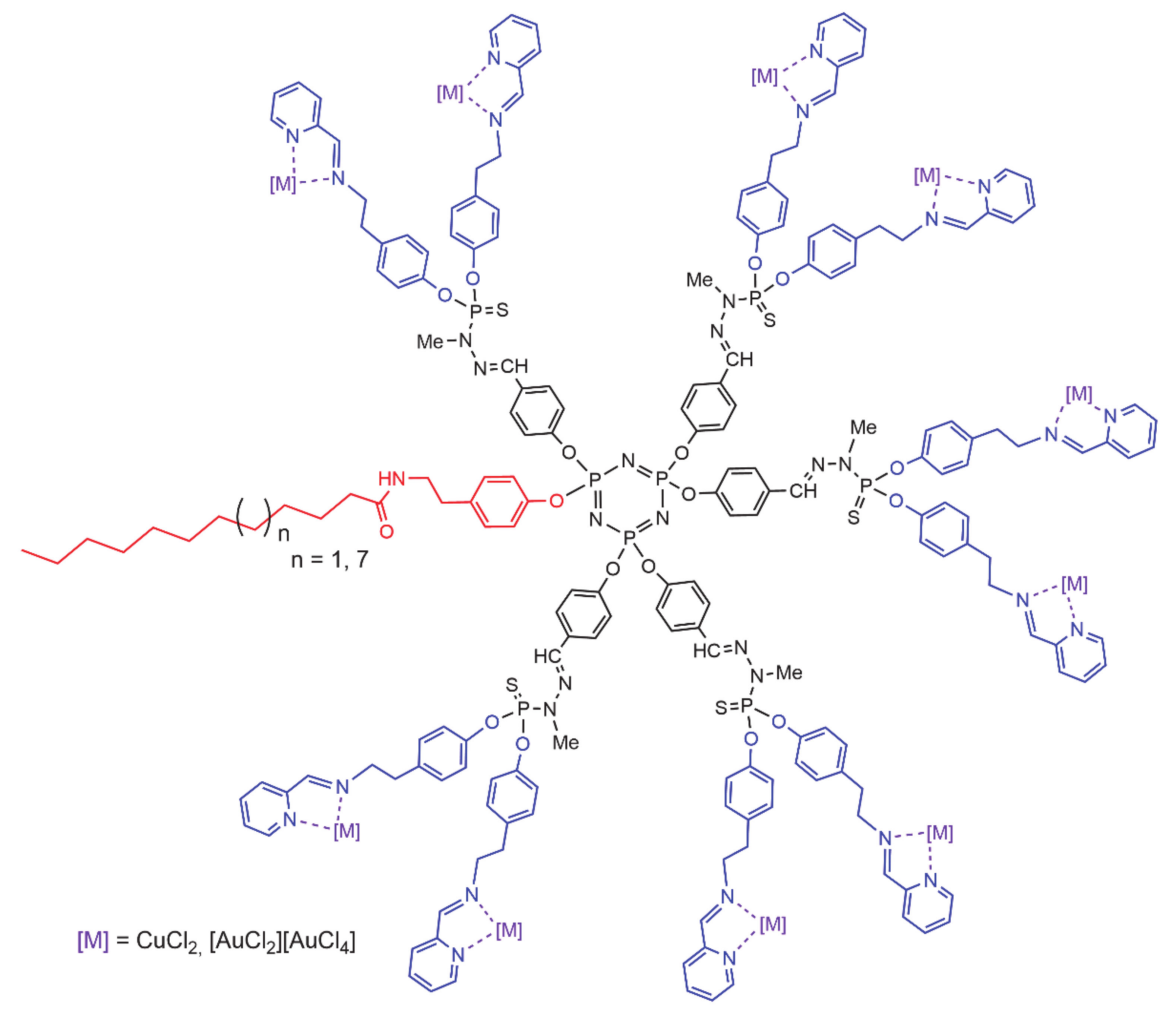
Dendrons having an alkyl chain at the core, and pyridine-imine functions complexing copper or gold on the surface.

Another recent result concerns a series of dendrons having an alkyl chain (C_11_H_23_ or C_17_H_35_) at the core, and pyridine-imine functions on the surface, suitable for the complexation of metals such as copper or gold [58]. The alkyl chain was grafted in the second step of the synthesis process, on a cyclotriphosphazene already functionalized with five equivalents of hydroxybenzaldehyde. The full structure of the first generation of this family of dendrons is shown in Figure 13. The corresponding plain dendrimers were synthesized before, and complexed with either copper or gold, or with a mixture of both metals. The copper complexes of the dendrimers were obtained by reaction of CuCl_2_. The third-generation dendrimer was found very efficient against a panel of human cancer cell lines, with IC_50_ values ranging 0.3−1.6 μM, whereas CuCl_2_ alone at the same concentration displayed no cytotoxicity [59]. An original mechanism of action of these dendrimers was proposed. The penetration in cells occurred by endocytosis, then the potent apoptosis activation could be related to a noticeable translocation of Bax (a proapoptotic protein) to the mitochondria, resulting in the release of AIF (Apoptosis Inducing Factor) into the cytosol, its translocation to the nucleus and a severe DNA fragmentation, without alteration of the cell cycle [60]. Later on, it was shown that replacing copper by gold, i.e., CuCl_2_ by [AuCl_2_][AuCl_4_] strongly increased the antiproliferative activities against both KB and HL-60 tumoral cell lines, showing IC_50_s in the low nanomolar range [61]. The dendrons shown in Figure 13 were tested against several aggressive breast cancer cell lines. It was shown that the cytotoxicity increased when reducing the length of the alkyl chain at the core. Furthermore, the replacement of copper by gold considerably increased the anti-proliferative activity [58], as was shown previously with the corresponding third generation dendrimers.

## 6. Conclusions

The simplicity of the synthesis and purification by routine column chromatography make AB_5_ derivatives of cyclotriphosphazene suitable starting reagents for the synthesis of particularly elaborated compounds. As two ways are usable for the synthesis of AB_5_ derivatives of N_3_P_3_ (one A followed by 5 B, or 5 B followed by 1 A) the process can be adapted depending on the sensitivity of one of the substituents, but in most cases a “me too” procedure is used. It means that, if a compound is synthesized in one way, analogous derivatives are synthesized in the same way, except if some problems occur at the purification step.

We have shown in this review the numerous properties of the AB_5_ derivatives of cyclotriphosphazene for the synthesis of diverse dendritic structures, such as layered dendrimers, dumbbell shape dendrimers, or Janus dendrimers. Dendrons based on AB_5_ derivatives of cyclotriphosphazene have been already used in very different fields ranging from nanomaterials to catalysis or sensors, and to different fields of biology, in particular, to fight against chronic or acute inflammations and against different types of cancers. It should be emphasized that most properties were observed with small dendritic structures of generations 0 or 1, to avoid the burying, and thus the inefficiency of the single function at the core. Despite the numerous works already carried out, there is still plenty of room for the synthesis of other dendritic structures based on AB_5_ derivatives of cyclotriphosphazene. Furthermore, we have also described a few examples concerning A_2_B_4_ derivatives of cyclotriphosphazene in which two functions at the core are modified, while preserving most of the properties of dendritic structures. One can also envisage the synthesis of ABC_4_ derivatives, having two different functions at the level of the core, potentially interesting in the fields of biology and nanomedicine, to incorporate specifically both a fluorophore and a targeting function, or whatever can be imagined.

Besides the synthesis of dendritic structures based on AB_5_ derivatives of cyclotriphosphazene, which is presently an active area of research, playing with the specific functionalization of the cyclotriphosphazene could also be used for the functionalization of other types of macromolecules. To the best of our knowledge, there is no example to date, except ill-defined polymeric species.

## Figures and Tables

**Table 1:** Number of references (and %) concerning AB_5_ derivatives of cyclotriphosphazene.

AB_5_ or A_5_B	Number of References ^1^	% ^2^
1 N/5 Cl	279	83.5
1 Cl/5 N	55	16.5
1 O/5 Cl	319	61.4
1 Cl/5 O	200	38.6
1 C/5 Cl	71	79.8
1 Cl/5 C	18	20.2
1 O/5 N	87	
1 N/5 O	95	
1 C/5 N	4	
1 C/5 O	29	

^1^ Source SciFinder^®^ on 17 June 2021; ^2^ % for AB_5_ compared to A_5_B.

## Sample Availability

No sample of compounds is available from the authors.

## References

[1] Liebig J. (1834). Nachtrag der redaction. Ann. Pharm..

[2] Gladstone J.H., Holmes J.D. (1864). On Chlorophosphuret of nitrogen, and its products of decomposition. J. Chem. Soc..

[3] De Jaeger R., Gleria M. (1998). Poly(organophosphazene)s and related compounds: Synthesis, properties and applications. Prog. Polym. Sci..

[4] Keat R., Shaw R.A. (1965). Phosphorus-Nitrogen compounds. Part IX. The reaction of dimethylamine with hexachlorocyclotriphosphazatriene: The replacement pattern and the structure of the products. J. Chem. Soc..

[5] Das S.K., Keat R., Shaw R.A., Smith B.C. (1965). Phosphorus-Nitrogen compounds. Part XVI. The reactions of hexachlorocyclotriphosphazatriene with t-butylamine. J. Chem. Soc..

[6] Das S.K., Keat R., Shaw R.A., Smith B.C. (1966). Phosphorus-Nitrogen compounds. Part XXII. The reactions of hexachlorocyclotriphosphazatriene with isopropylamine. J. Chem. Soc. A.

[7] Harris P.J., Schwalke M.A., Liu V., Fisher B.L. (1983). Phosphazenes.2. Synthesis of ketone-substituted and enol-substituted cyclotriphosphazenes. Inorg. Chem..

[8] Allcock H.R., Brennan D.J., Graaskamp J.M., Parvez M. (1986). Synthesis and molecular structure of methylsilane cyclotriphosphazenes and methylsiloxane cyclotriphosphazenes. Organometallics.

[9] Allcock H.R., Nelson C.J., Coggio W.D. (1991). Synthesis and reactivity of cyclotriphosphazene bearing reactive silane functionalities: Novel derivatives via hydrosilylation reactions. Organometallics.

[10] Diefenbach U., Kretschmann M., Stromburg B. (1996). Phosphazenes with (2-pyridylmethylamino) groups.1. Syntheses and crystal structures of pentaphenoxy(2-pyridylmethylamino)cyclotriphosphazene and its copper(II) nitrate, platinum(II) chloride, and cobalt(II) nitrate complexes. Chem. Ber..

[11] Davidson R.J., Ainscough E.W., Brodie A.M., Jameson G.B., Waterland M.R., Moubaraki B., Murray K.S., Gordon K.C., Horvath R., Jameson G.N.L. (2013). An iron(II) spin crossover grafted cyclotriphosphazene. Polyhedron.

[12] Scopelianos A.G., Obrien J.P., Allcock H.R. (1980). Cyclic and polymeric phosphazenes with carborane side groups X-ray crystal structure of a carborane-substituted cyclophosphazene. J. Chem. Soc. Chem. Commun..

[13] Allcock H.R., Scopelianos A.G., Obrien J.P., Bernheim M.Y. (1981). Synthesis and structure of carborane-substituted cyclic and polymeric phosphazenes. J. Am. Chem. Soc..

[14] Kato F., Chandra A., Tokita M., Asano H., Shimomoto H., Ihara E., Hayakawa T. (2018). Self-assembly of hierarchical structures using cyclotriphosphazene-containing poly(substituted methylene) block copolymers. ACS Macro Lett..

[15] Caminade A.-M., Turrin C.-O., Laurent R., Ouali A., Delavaux-Nicot B. (2011). Dendrimers: Towards Catalytic, Material and Biomedical Uses.

[16] Caminade A.M., Ouali A., Laurent R., Turrin C.O., Majoral J.P. (2016). Coordination chemistry with phosphorus dendrimers. Applications as catalysts, for materials, and in biology. Coord. Chem. Rev..

[17] Slany M., Bardaji M., Casanove M.J., Caminade A.M., Majoral J.P., Chaudret B. (1995). Dendrimer surface-chemistry—Facile route to polyphosphines and their gold complexes. J. Am. Chem. Soc..

[18] Sournies F., Crasnier F., Graffeuil M., Faucher J.P., Lahana R., Labarre M.C., Labarre J.F. (1995). Spherical Cyclophosphazene Dendrimers to the 5th Generation. Angew. Chem. Int. Ed..

[19] Maraval V., Caminade A.M., Majoral J.P., Blais J.C. (2003). Dendrimer design: How to circumvent the dilemma of a reduction of steps or an increase of function multiplicity?. Angew. Chem. Int. Ed..

[20] Kolb H.C., Finn M.G., Sharpless K.B. (2001). Click chemistry: Diverse chemical function from a few good reactions. Angew. Chem. Int. Ed..

[21] Franc G., Kakkar A.K. (2010). “Click’’ methodologies: Efficient, simple and greener routes to design dendrimers. Chem. Soc. Rev..

[22] Sharma R., Zhang I., Abbassi L., Rej R., Maysinger D., Roy R. (2015). A fast track strategy toward highly functionalized dendrimers with different structural layers: An “onion peel approach”. Polym. Chem..

[23] Abbassi L., Chabre Y.M., Kottari N., Arnold A.A., Andre S., Josserand J., Gabius H.J., Roy R. (2015). Multifaceted glycodendrimers with programmable bioactivity through convergent, divergent, and accelerated approaches using polyfunctional cyclotriphosphazenes. Polym. Chem..

[24] Launay N., Caminade A.M., Lahana R., Majoral J.P. (1994). A general synthetic strategy for neutral phosphorus-containing dendrimers. Angew. Chem. Int. Ed..

[25] Krishna T.R., Parent M., Werts M.H.V., Moreaux L., Gmouh S., Charpak S., Caminade A.M., Majoral J.P., Blanchard-Desce M. (2006). Water-soluble dendrimeric two-photon tracers for in vivo imaging. Angew. Chem. Int. Ed..

[26] Mongin O., Rouxel C., Robin A.C., Pla-Quintana A., Krishna T.R., Recher G., Tiaho F., Caminade A.M., Majoral J.P., Blanchard-Desce M., Heckman E.M., Singh T.B., Yoshida J. (2008). Brilliant organic nanodots: Novel nano-objects for bionanophotonics. Nanobiosystems: Processing, Characterization, and Applications.

[27] Caminade A.M., Laurent R., Delavaux-Nicot B., Majoral J.P. (2012). “Janus” dendrimers: Syntheses and properties. New J. Chem..

[28] Hameau A., Fuchs S., Laurent R., Majoral J.P., Caminade A.M. (2011). Synthesis of dye/fluorescent functionalized dendrons based on cyclotriphosphazene. Beilstein J. Org. Chem..

[29] Fuchs S., Pla-Quintana A., Mazeres S., Caminade A.M., Majoral J.P. (2008). Cationic and Fluorescent “Janus” Dendrimers. Org. Lett..

[30] Martinez-Ferrero E., Franc G., Mazeres S., Turrin C.O., Boissiere U., Caminade A.M., Majoral J.P., Sanchez C. (2008). Optical properties of hybrid dendritic-mesoporous titania nanocomposite films. Chem. A Eur. J..

[31] Riegert D., Pla-Quintana A., Fuchs S., Laurent R., Turrin C.O., Duhayon C., Majoral J.P., Chaumonnot A., Caminade A.M. (2013). Diversified Strategies for the Synthesis of Bifunctional Dendrimeric Structures. Eur. J. Org. Chem..

[32] Riegert D., Bareille L., Laurent R., Majoral J.P., Caminade A.M., Chaumonnot A. (2016). Silica Functionalized by Bifunctional Dendrimers: Hybrid Nanomaterials for Trapping CO_2_. Eur. J. Inorg. Chem..

[33] de Jong E.R., Deloch N., Knoll W., Turrin C.O., Majoral J.P., Caminade A.M., Koper I. (2015). Synthesis and characterization of bifunctional dendrimers: Preliminary use for the coating of gold surfaces and the proliferation of human osteoblasts (HOB). New J. Chem..

[34] Keller M., Colliere V., Reiser O., Caminade A.M., Majoral J.P., Ouali A. (2013). Pyrene-Tagged Dendritic Catalysts Noncovalently Grafted onto Magnetic Co/C Nanoparticles: An Efficient and Recyclable System for Drug Synthesis. Angew. Chem. Int. Ed..

[35] Asri H., Dautel O., Ouali A. (2020). Terpyridine-Ru Complexes Noncovalently Supported on Cobalt Magnetic Nanoparticles for Nitroarene Transfer Hydrogenation. ACS Appl. Nano Mater..

[36] Zapsas G., Patil Y., Gnanou Y., Ameduri B., Hadjichristidis N. (2020). Poly(vinylidene fluoride)-based complex macromolecular architectures: From synthesis to properties and applications. Prog. Polym. Sci..

[37] Folgado E., Guerre M., Mimouni N., Colliere V., Bijani C., Ching K.M.C., Caminade A.M., Ladmiral V., Ameduri B., Ouali A. (2019). pi-Stacking Interactions of Graphene-Coated Cobalt Magnetic Nanoparticles with Pyrene-Tagged Dendritic Poly(Vinylidene Fluoride). ChemPlusChem.

[38] Caminade A.-M. (2017). Phosphorus dendrimers for nanomedicine. Chem. Commun..

[39] Franc G., Mazeres S., Turrin C.O., Vendier L., Duhayon C., Caminade A.M., Majoral J.P. (2007). Synthesis and properties of dendrimers possessing the same fluorophore(s) located either peripherally or off-center. J. Org. Chem..

[40] Loup C., Zanta M.A., Caminade A.M., Majoral J.P., Meunier B. (1999). Preparation of water-soluble cationic phosphorus-containing dendrimers as DNA transfecting agents. Chem. A Eur. J..

[41] Kazmierczak-Baranska J., Pietkiewicz A., Janicka M., Wei Y.Q., Turrin C.O., Majoral J.P., Nawrot B., Caminade A.M. (2010). Synthesis of a Fluorescent Cationic Phosphorus Dendrimer and Preliminary Biological Studies of Its Interaction with DNA. Nucleosides Nucleotides Nucleic Acids.

[42] Qiu J., Chen L., Zhan M., Laurent R., Bignon J., Mignani S., Shi X., Caminade A.-M., Majoral J.-P. (2021). Facile Synthesis of Amphiphilic Fluorescent Phosphorus Dendron-Based Micelles as Antiproliferative Agents: First Investigations. Bioconjug. Chem..

[43] Poupot M., Griffe L., Marchand P., Maraval A., Rolland O., Martinet L., L’Faqihi-Olive F.E., Turrin C.O., Caminade A.M., Fournie J.J. (2006). Design of phosphorylated dendritic architectures to promote human monocyte activation. FASEB J..

[44] Griffe L., Poupot M., Marchand P., Maraval A., Turrin C.O., Rolland O., Metivier P., Bacquet G., Fournie J.J., Caminade A.M. (2007). Multiplication of human natural killer cells by nanosized phosphonate-capped dendrimers. Angew. Chem. Int. Ed..

[45] Poupot M., Turrin C.O., Caminade A.M., Fournie J.J., Attal M., Poupot R., Fruchon S. (2016). Poly(phosphorhydrazone) dendrimers: Yin and yang of monocyte activation for human NK cell amplification applied to immunotherapy against multiple myeloma. Nanomed. Nanotechnol. Biol. Med..

[46] Hayder M., Poupot M., Baron M., Nigon D., Turrin C.O., Caminade A.M., Majoral J.P., Eisenberg R.A., Fournie J.J., Cantagrel A. (2011). A Phosphorus-Based Dendrimer Targets Inflammation and Osteoclastogenesis in Experimental Arthritis. Sci. Transl. Med..

[47] Hayder M., Varilh M., Turrin C.O., Saoudi A., Caminade A.M., Poupot R., Liblau R.S. (2015). Phosphorus-Based Dendrimer ABP Treats Neuroinflammation by Promoting IL-10-Producing CD4(+) T Cells. Biomacromolecules.

[48] Rolland O., Griffe L., Poupot M., Maraval A., Ouali A., Coppel Y., Fournie J.J., Bacquet G., Turrin C.O., Caminade A.M. (2008). Tailored control and optimisation of the number of phosphonic acid termini on phosphorus-containing dendrimers for the ex-vivo activation of human monocytes. Chem. A Eur. J..

[49] Portevin D., Poupot M., Rolland O., Turrin C.O., Fournie J.J., Majoral J.P., Caminade A.M., Poupot R. (2009). Regulatory activity of azabisphosphonate-capped dendrimers on human CD4(+) T cell proliferation enhances ex-vivo expansion of NK cells from PBMCs for immunotherapy. J. Transl. Med..

[50] Soler-Illia G., Rozes L., Boggiano M.K., Sanchez C., Turrin C.O., Caminade A.M., Majoral J.P. (2000). New mesotextured hybrid materials made from assemblies of dendrimers and titanium(IV)-oxo-organo clusters. Angew. Chem. Int. Ed..

[51] Ielasi F., Ledall J., Anes A.P., Fruchon S., Caminade A.M., Poupot R., Turrin C.O., Blanzat M. (2016). Influence of PPH dendrimers’ surface functions on the activation of human monocytes: A study of their interactions with pure lipid model systems. Phys. Chem. Chem. Phys..

[52] Rolland O., Turrin C.O., Bacquet G., Poupot R., Poupot M., Caminade A.M., Majoral J.P. (2009). Efficient synthesis of phosphorus-containing dendrimers capped with isosteric functions of amino-bismethylene phosphonic acids. Tetrahedron Lett..

[53] Ledall J., Fruchon S., Garzoni M., Pavan G.M., Caminade A.M., Turrin C.O., Blanzat M., Poupot R. (2015). Interaction studies reveal specific recognition of an anti-inflammatory polyphosphorhydrazone dendrimer by human monocytes. Nanoscale.

[54] Caminade A.M., Fruchon S., Turrin C.O., Poupot M., Ouali A., Maraval A., Garzoni M., Maly M., Furer V., Kovalenko V. (2015). The key role of the scaffold on the efficiency of dendrimer nanodrugs. Nature Commun..

[55] Hayder M., Garzoni M., Bochicchio D., Caminade A.-M., Couderc F., Ong-Meang V., Davignon J.-L., Turrin C.-O., Pavan G.M., Poupot R. (2018). Three-Dimensional Directionality Is a Pivotal Structural Feature for the Bioactivity of Azabisphosphonate-Capped Poly(PhosphorHydrazone) Nanodrug Dendrimers. Biomacromolecules.

[56] Fruchon S., Bellard E., Beton N., Goursat C., Oukhrib A., Caminade A.-M., Blanzat M., Turrin C.-O., Golzio M., Poupot R. (2019). Biodistribution and Biosafety of a Poly(Phosphorhydrazone) Dendrimer, an Anti-Inflammatory Drug-Candidate. Biomolecules.

[57] Jebbawi R., Oukhrib A., Clement E., Blanzat M., Turrin C.-O., Caminade A.-M., Lacoste E., Fruchon S., Poupot R. (2020). An Anti-Inflammatory Poly(PhosphorHydrazone) Dendrimer Capped with AzaBisPhosphonate Groups to Treat Psoriasis. Biomolecules.

[58] Chen L., Fan Y., Qiu J., Laurent R., Li J., Bignon J., Mignani S., Caminade A.-M., Shi X., Majoral J.-P. (2020). Potent Anticancer Efficacy of First-In-Class Cu-II and Au-III Metaled Phosphorus Dendrons with Distinct Cell Death Pathways. Chem. A Eur. J..

[59] El Brahmi N., El Kazzouli S., Mignani S.M., Essassi E., Aubert G., Laurent R., Caminade A.M., Bousmina M.M., Cresteil T., Majoral J.P. (2013). Original Multivalent Copper(II)-Conjugated Phosphorus Dendrimers and Corresponding Mononuclear Copper(II) Complexes with Antitumoral Activities. Mol. Pharm..

[60] Mignani S., El Brahmi N., Eloy L., Poupon J., Nicolas V., Steinmetz A., El Kazzouli S., Bousmina M.M., Blanchard-Desce M., Caminade A.M. (2017). Anticancer copper(II) phosphorus dendrimers are potent proapoptotic Bax activators. Eur. J. Med. Chem..

[61] Mignani S.M., El Brahmi N., El Kazzouli S., Laurent R., Ladeira S., Caminade A.-M., Pedziwiatr-Werbicka E., Szewczyk E.M., Bryszewska M., Bousmina M.M. (2017). Original Multivalent Gold(III) and Dual Gold(III)-Copper(II) Conjugated Phosphorus Dendrimers as Potent Antitumoral and Antimicrobial Agents. Mol. Pharm..

